# Security in IoMT Communications: A Survey

**DOI:** 10.3390/s20174828

**Published:** 2020-08-26

**Authors:** Dimitris Koutras, George Stergiopoulos, Thomas Dasaklis, Panayiotis Kotzanikolaou, Dimitris Glynos, Christos Douligeris

**Affiliations:** 1 Department of Informatics, University of Piraeus, 80, M. Karaoli & A. Dimitriou St., 18534 Piraeus, Greece; yiorgos.stergiopoulos@gmail.com (G.S.); dasaklis@unipi.gr (T.D.); cdoulig@unipi.gr (C.D.); 2 CENSUS S.A., I. Gkoura 16, 54352 Thessaloniki, Greece; dimitris@census-labs.com

**Keywords:** IoT communication protocols, internet of medical things, security, perception-network-application layer

## Abstract

The Internet of Medical Things (IoMT) couples IoT technologies with healthcare services in order to support real-time, remote patient monitoring and treatment. However, the interconnectivity of critical medical devices with other systems in various network layers creates new opportunities for remote adversaries. Since most of the communication protocols have not been specifically designed for the needs of connected medical devices, there is a need to classify the available IoT communication technologies in terms of security. In this paper we classify IoT communication protocols, with respect to their application in IoMT. Then we describe the main characteristics of IoT communication protocols used at the perception, network and application layer of medical devices. We examine the inherent security characteristics and limitations of IoMT-specific communication protocols. Based on realistic attacks we identify available mitigation controls that may be applied to secure IoMT communications, as well as existing research and implementation gaps.

## 1. Introduction

The term Internet of Things (IoT) refers to a wide range of interconnected objects and devices that harvest information from the environment through sensors, analyze it and act back on the physical world through actuators [1]. Although similar to modern cyberphysical systems, IoT incorporates a wide area of applications in various sectors, including smart energy grids, industrial control systems, healthcare, transportation, home appliances and wearables [2]. Despite the obvious operational and functional benefits, the integration of IoT technologies has also led to new attack opportunities for remote adversaries. Recent real-world incidents and proof of concept attacks have demonstrated the rise of IoT-enabled attacks in all these sectors [3]. Indeed, the increase of interconnectivity and interoperability of previously isolated systems create new attack paths for remote adversaries.

Since IoT technologies are utilized in various sectors with different security requirements and needs, they are not always designed having in mind the specific threat landscape of a particular sector. In the healthcare sector, IoT devices, also known as Internet of Medical Things (IoMT), may support core functions of healthcare or health-related services. IoMT allows the coupling of IoT communication protocols with medical systems and devices, in order to support real-time, remote patient monitoring and treatment. For example, smart hospitals integrate IoMT to “provide optimised and the automated processes built on an Information and Communication Technologies (ICT) environment of interconnected assets, particularly based on IoT, to improve existing patient care procedures and introduce new capabilities” [4]. Since most of the communication protocols have not been specifically designed for the needs of connected medical devices, there is a need to evaluate the available IoT communication technologies in terms of security, in the context of medical devices. To perform this evaluation we use a classification based on the three layers of the IoT communication protocols [5], the perception, network and application layers as described below. Each layer effectively contains specific types of protocols and ways for information exchange, much like the Open Systems Interconnection (OSI) layers.

*Perception layer*: It is the lowest layer where signal measurement and transmission can take place. It is the layer closest to the hardware Medium Access Control (MAC) layer [6].

*Network layer*: It is similar to the OSI network layer. It is responsible for the communication, the interconnection and the transport of data packets among devices through the network.

*Application layer*: It is the top layer in the three-layered IoT architecture. It corresponds to the session and application layer in the OSI model. This layer provides application and data control services. It can mostly be affected by the type of data travelling through the IoT network [6].

Due to the highly sensitive nature of the data being processed in IoMT, interconnections between medical devices must be secured and always available. In addition, data integrity, confidentiality and availability are of paramount importance for the medical data shared in the hospital’s network. Various operational and interoperability challenges should also be taken into account. For example, the multitude of medical assets used throughout the healthcare ecosystem calls for the integration of different technologies to be used under the same IoMT ecosystem.

### 1.1. Research Motivation

The recent literature includes surveys that provide various taxonomies of IoT communication protocols [7,8,9], including surveys that pay special attention to the security aspects of an IoT system [10]. Other studies provide a generic analysis of healthcare systems and relevant devices. In [11] the authors analyse the various prerequisites for successfully integrating medical systems in an IoT network. Other works focus on communication protocols used in IoMT systems. For example, Javdani et al. [12] group IoT communication protocols within the three-layer IoT architecture and propose the inclusion of these protocols in IoMT networks. Fotouhi et al. [13] also present some IoT communication protocols that are popular in IoMT. They also present some key security characteristics of the communication protocols. Unfortunately, the authors fail to provide how the various protocol security issues may affect or interact with an IoMT system network.

Despite the significant benefits of the studies above, there is a clear lack of a systematic taxonomy of IoMT-specific communication protocols regarding (a) their relevant context-specific security challenges, (b) existing security solutions and (c) the various research and implementation gaps as derived by the IoMT ecosystem.

### 1.2. Contribution—Paper Structure

The goal of this survey is to provide a detailed classification of the IoMT-specific communication protocols used throughout various medical devices within the healthcare ecosystem. In particular, we employ different drivers for classifying the selected literature such as: (a) the various layers of the generic IoT architecture; (b) the categories of the medical devices that utilise these protocols; (c) the inherent security characteristics and/or vulnerabilities of the IoMT-specific communication protocols and (d) possible mitigation controls. Towards this direction, the main contributions of the paper are:

1. We provide an exhaustive list of IoMT-specific communication protocols. In Section 3 we present a detailed classification of IoT communication protocols based on the three-layered approach (Section 3.1) and also based on the specific categories of IoMT devices (Section 3.2).
2. Based on the above taxonomy, we present, for all the IoMT-specific communication protocols identified, their inherent security characteristics (Section 4.1), as well as their security weaknesses and relevant attacks, as derived by the literature (Section 4.2). In addition, based on the IoMT protocol taxonomy, we discuss mitigation controls for IoMT-specific communication protocols that take into consideration the limitations of the medical ecosystem (Section 4.3).
3. Having in mind the current security status of the IoMT communication protocols described above, we discuss real case attack scenarios against medical devices (Section 5.1). In addition, we provide a comparative assessment of the various characteristics of the IoMT-specific communication protocols. Based on various use case scenarios, in Section 5.2 we provide a suitability assessment of the aforementioned protocols (whose protocol would be more suitable for the various types of medical devises, the underlying infrastructure and technologies used).
4. Finally, in Section 6 we discuss open issues and challenges for IoMT protocol security.

## 2. Research Methodology

**Figure 1:**
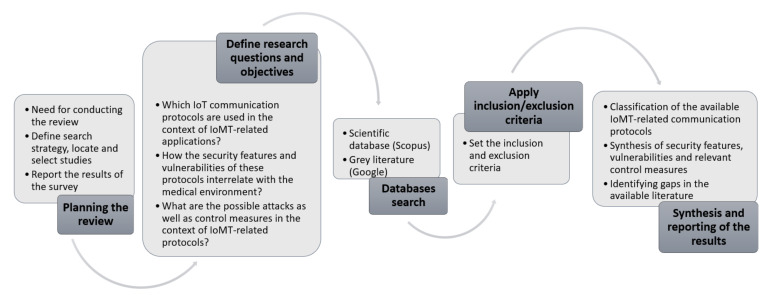
Steps of the overall research methodology.

This section covers the methodological aspects of our review protocol. For conducting our review we have used various features from the approach presented in [14]. As seen in Figure 1, our review protocol consists of five steps: (1) Planning the review (2) Defining research questions (3) Database search (4) Applying inclusion and exclusion criteria and (5) Synthesising and reporting the results of the survey.

Our overall survey process relies on several predefined research questions relevant to IoMT-specific communication protocols. Based on these research questions, we have conducted an extensive research addressing the various technical/functional/security challenges of the IoMT-related communication protocols. Our main research questions (RQ) are as follows:

***RQ1*:**  Which IoT communication protocols are used in the context of IoMT?
***RQ2*:**  What are the security features and possible vulnerabilities of these protocols and how do they interrelate with the medical environment?
***RQ3*:**  What are the possible attacks as well as the likely available control measures in the context of IoMT-related communication protocols?

Our overall search strategy relied on the Scopus scientific database for locating relevant papers. For broadening our analysis, we have also evaluated the first 100 hits from Google, particularly for taking into account relevant grey literature sources (such as committee and policy reports, industry-related reports, etc.). We evaluated the eligibility of the retrieved literature based on a set of inclusion/exclusion criteria. Since there is sufficient overlapping between IoT and IoMT communication protocols and most IoT protocols are used in healthcare and medical applications, we searched for two main streams of literature in the Scopus database (peer-reviewed research articles, conference proceedings, book chapters, review papers, short surveys, etc.). The first stream helped us identify research papers addressing issues of communication protocols within healthcare/medical applications. After having defined these protocols (IoMT-specific), we extensively searched the IoT-related literature for deriving various technical, functional and security characteristics of the finally established IoMT-specific communication protocols. It should be noted, also, that we have included in our analysis only English-written papers/reports. We took into account for our analysis 166 research papers and 37 reports. In Table 1, we present the key-words we used for locating our sources (both scientific and grey literature).

For reporting the results of our study in a sound and comprehensive manner, we have used various ways to synthesise the available literature. For example, we present two distinct categorisations of the available IoMT-specific communication protocols based on (a) the underlying layers of the IoT architecture and (b) their application in various categories of medical devices. In addition, we synthesise the various security issues of the IoMT-specific communication protocols by establishing a link between possible vulnerabilities and attack scenarios and relevant control measures.

## 3. Classification of IoT Protocols Used in Medical Devices

The IoMT communication protocols support the interaction of near-patient medical devices with other medical-related Information Technology (IT) systems. IoMT devices may utilise sensors and/or actuators to monitor and treat patients in real-time. In the first case, sensors may acquire data from patients, using some IoT communication technology at the perception layer. Then, network layer communication protocols may transmit this information to IT systems after they have transformed it to a form suitable for a particular medical application. This information may then trigger real-time patient treatment. In the second case, actuators connected to patients may support the real-time provisioning of medical treatment. Smart IoMT devices may process the information received from presumably trusted communication channels, in order to modify medicine treatment protocols (e.g., modify the dosage to be injected).

Obviously, securing the connectivity of IoMT systems at all layers is a high priority, since the modification, disclosure or unavailability of the relevant information may be life threatening [12,15]. Since IoMT devices are a subset of the IoT ecosystem, their communications rely on IoT-specific protocols. However, not all of the IoT communication protocols have been applied in IoMT devices. First we will provide a brief summary of IoT communication protocols that are applied in IoMT communications (Section 3.1). Then, we will categorise these protocols based on their application in various categories of medical devices (Section 3.2).

### 3.1. Medical-Specific IoT Communication Protocols

We briefly describe the medical-specific IoT communication protocols (called hereafter IoMT protocols for short) based on the layered IoT architecture of [16]; the perception, the network and the application layer. Note that two more sublayers between those main layers exist, mainly the adaption layer and the transport layer [17]. The protocols that transact between the perception and the network layer are integrated into the adaption layer. In addition, the protocols that carry the network information from the network to the application layer are included in the transport layers [18].

#### 3.1.1. Perception Layer

Most of the perception layer protocols are based on or implement the IEEE 802.15.4 standard [19]. The IEEE 802.15.4 is a standard for ultra low complexity, cost and power consumption. It supports wireless connectivity of relatively low data rate and it mainly focuses on inexpensive, low spectrum IoT devices. IEEE 802.15.4 describes the specifications for the physical (PHY) and the MAC layers, for various types of devices (fixed, portable or moving devices), having very limited battery consumption requirements or even having no battery at all [19]. It adopts a wide-band physical layer using the Direct Sequence Spread Spectrum technique. It supports physical layer operations in three frequency bands: (a) the 868 MHz band, available in Europe, (b) the 915 MHz band, available in US, and (c) the 2.4 GHz ISM band, which is the unlicensed band available globally. Twenty-seven channels are supported across these three bands. The physical layer of the IEEE 802.15.4 is responsible for various low level functions including among others, data transfer and receipt, energy detection of current channel, link quality and clear channel assessment. The MAC (data-link) layer of IEEE 802.15.4 is responsible for functionalities including, joining and leaving the Personal Area Network (PAN); Carrier Sense Multiple Access with Collision Avoidance (CSMA-CA) for channel access; Guaranteed Time Slot (GTS) transmissions; Reliability of link establishment between peer MAC entities; Controlling beacon transmissions for a coordinator; Ensuring synchronisation to the beacons [20].

Various IoMT related protocols are compatible with IEEE 802.15.4, such as ZigBee, LowPan and ISA 100.11a [21]. The following perception layer protocols have been utilised by healthcare systems to gather clinical information from the sensors.

##### Infrared

The infrared technology proposed by the Infrared Data Association (IrDA) uses infrared light for short-range communication [22]. IrDA provides guidance for wireless infrared communication protocols that work up to a few meters away with directed, targeted infrared beams. The IrDA protocol stack is comprised of three mandatory layers: the physical, the link access (IrLAP) and the link management layer (IrLMP). The IrLAP protocol provides basic link layer connection between pairs of devices and is based on the High-level Data Link Control (HDLC) standard for connection establishment and data transfer [23]. The IrLMP protocol provides a way for multiple entities within pairs of IrDA devices to simultaneously and independently use the single IrLAP connection and allows for service discovery by pairing entities [24]. Device discovery processes the receiving (within range) device’s address along with its IrLAP version and some discovery information specified by the IrLMP layer. Within the medical domain, IrDA is used in temperature sensors in thermometers and cameras. The infrared-based thermal imaging technology is also used to take pictures of a body and find its local temperature [25,26].

##### RFID

Radio Frequency Identification (RFID) is a wireless object identification technology that uses radio frequency signals for very short range communications. Two are the main entities in RFID communications: a reading device (the RFID reader) and the radio transponder device (called the RF tag). The RF reader stores data and has remote distance reading features. The RF tag will first receive a message from the reader and will then respond by sending some identification (and/or some additional) information back to the reader. Usually, a unique serial number serves as the identification information, while additional information may be product related, such as stock or batch number, production date, etc. There exist two main technologies for the RFID tags: active reading tags and passive reading tags. The active reader tags are power-driven, use high-frequency bands and are relatively high-priced; on the other hand, the passive tags usually operate on lower frequencies and do not have an internal power source. The following frequency ranges are commonly used in RF communications: (a) Low Frequency (LF, 125 kHz), (b) High Frequency (HF, 13.56 MHz), (c) Ultra High Frequency (UHF, 433 MHz, 860–960 MHz) and (d) Microwave (2.45 GHz, 5.8 GHz) [8,10,18].

Autonomous RFID tags placed inside or in close proximity to a patient’s body are key enabling technologies to develop body area healthcare systems which are fully transparent to the patient [27]. In addition, passive RFID tag may be applied for the ambient monitoring of patients’ environment (e.g., to detect environmental changes such as modification in physical or chemical parameters). RFID has also been used for physical access control [8,28]; for example, hospitals may use RFID cards to secure physical access. Another example of RFID use is presented in [7], where authors utilise tags and sensors to provide sensitive temperature monitoring for drug storage to adjust a suitable temperature for each type of drug accordingly [29].

##### NFC

Near Field Communication (NFC) protocol supports very short range communication among devices (in the range of few centimeters at most). NFC is standardised in ECMA-340 and ISO/IEC 18092. Based on the inductive coupling between the transmitting and receiving device, its short range and high frequency (13.56 MHz) technology allows data transfer rates up to 424 Kbps [7,12]. As in the case of RFID, the NFC also operates in passive and active modes. In the case of a passive mode, one device is active and generates an RF field; the passive target device is woken up by using the energy generated by the active one. NFC is used for easy and low-cost connection among IoT devices. Most of the time, this protocol is used for authentication purposes; for example, it has to check whether an IoT device is registered in an authentication server, as well as to check whether the user is registered in the server.

Freudenthal et al. [30] examined the use of the NFC signals for medical devices inside a human body (e.g., ingestible or implantable sensors). Applying NFC within implanted or ingested medical devices presents various biocompatibility challenges. Note, however, that NFC-enabled medical devices do not necessarily require a battery or external electrical connections for their custom operation [30]. Freudenthal et al. also mention a field-powered experimental implantable ECG (electrocardiograph) [31].

##### Bluetooth/BLE

Bluetooth is a widespread wireless communication technology based on the IEEE 802.15.1 standard. It is suitable for low-power and low-cost devices and it is operating at the 2.4 GHz band. Bluetooth can support star topology Personal Area Networks (PAN), with lower power consumption, low setup time and with unlimited number of nodes [8]. It is suitable for data transmission between mobile devices over a short range, which, based on the version may vary; for example, in version 2.1 it supports a maximum indoor range up to 100 m, while in version 5 (BLE), it supports a range up to 400 m. The data rate in various versions of the Bluetooth ranges up to 2 Mbps [12,13]. Its ultra low power version is known as Bluetooth Low Energy (BLE) or Bluetooth Smart [10]. In its newer version (BLE 5.0) it supports 2 Mbps data transmission, leading to reduced power consumption due to the overall decrease in the transmission time. The reduced radio transmission time also leads to improved wireless coexistence. BLE provides the highest data rate (even at the original 1 Mbps data rate) when compared to other low-power wireless protocols, such as ZigBee or Z-Wave discussed below. In comparison with NFC, Bluetooth consumes more power, while it requires device pairing [8,10,18,32].

BLE is used in IoT networks involving battery operated, low power and low cost devices. It enables devices to join the network in a relatively short time and form simple device to device links or star networks. It is used for IoMT applications that require short distance communication, low latency and low bandwidth targeting applications like Human Interface Devices (HID), sports and fitness monitors and portable medical devices [33,34].

##### Z-Wave

Z-wave is a low-power wireless MAC protocol developed by Zensys. It can be used to set up mesh topology networks and it supports two types of devices, controlling and slave devices [8,32]. A Z-Wave network may host up to 232 nodes and may cover distances up to 32 m using point to point communications. It operates at 900 MHz and supports a transmission rate of 40 kbps.

Z-wave is suitable to support short messaging among IoT devices utilised for light, energy and healthcare control. Being a very low bandwidth and half duplex protocol, it is not a suitable choice for transferring a larger amount of data (e.g., streaming data). Thus, it is used in wireless home automation to connect 30–50 nodes and for IoT communications, especially in a smart home (smart locks, smart wearables and smart sensor control), healthcare areas and small commercial domains [18].

It is important to mention that some of the protocols mentioned in the perception layer like BLE and Z-Wave may also be found at the network layer [35].

##### UWB

Built over the IEEE 802.15.3 standard, the primary goal of the Ultra-Wideband (UWB) protocol is to support high speed and short range indoor wireless communications. The data rate of UWB may vary from 110 up to 480 Mbps, making it suitable for several applications of higher demand, like audio or video home networking applications. Due to its high bandwidth, UWB could also act as a wireless cable replacement, particularly for the high speed serial buses such as USB 2.0 and IEEE 1394. Various UWB regulatory initiatives exist worldwide. In February 2002 the Federal Communications Commission (FCC) ordered the frequency allocation for UWB in the United States. On February 2007, the EU Commission released its “Commission Decision” regarding the implementation of an UWB radio regulatory framework applicable to all European Union member states. UWB presents significant differences when compared to conventional radio transmissions. While other wireless technologies transmit information by varying the power level, frequency and/or phase of a sinusoidal wave, UWB transmits information by creating radio energy at specific time intervals and occupying a large bandwidth, thus enabling time or pulse-position modulation [36].

Due to its low power and high precision, UWB is suitable for real-time applications in RF sensitive environments, such as hospitals. Fotouhi et al. [37] propose UWB for medical systems, since high signal attenuation when communicating with an implanted sensor requires a protocol that transcends channel limitations. A typical use is to transmit signals from sensors to a microcontroller [7]. For example, the electrocardiogram procedure requires a short distance communication technology and UWB, among other protocols, has been used for this purpose [12,15,38].

#### 3.1.2. Network Layer

The network layer includes hardware like gateways, routers and access points and deals with Internet Protocol (IP) addressing (subneting) and other network capabilities [9]. Since in healthcare, privacy sensitive data are transferred by such network devices, network security is a primary concern [39]. In IoMT, network layer security must deal with trust management, confidentiality, integrity, authentication and protection from denial of service attacks [17]. Most of the protocols of this layer are based on the IEEE 802.15 standard [40]. WiFi and ZigBee are the most common protocols for IoMT at this layer. Bluetooth is also used but less often, since it cannot cover large areas like hospitals. Other protocols used in medical systems are [32] LoRaWAN and 6LoWPAN for the association with Wireless Sensor Networks. Typical cellular communication technologies (such as GPRS or 3/4/5 G) can then be used for remote data transfer and communication.

##### WiFi

Wireless Fidelity (WiFi) is a middle range (up to 100 m) protocol, which is based on the IEEE 802.11 family of standards. WiFi is widely used for handheld devices and for local area networking, to support Internet access for multiple devices. Currently, the most common WiFi standard used is 802.11 n, which may support throughput rate of hundreds of Mbps, making it suitable for file transfer. However, it is not always suitable for IoT applications, due to its relatively high power consumption [10,41]. Indeed, the initial WiFi standards not only require high power consumption, but they also have a significant frame overhead. To cover this gap, the IEEE 802.11 working group initiated 802.11ah task group, in order to develop a standard that is more friendly to devices with low-power consumption and low frame overhead needs, such as sensors and motes.

Several authors have proposed the use of WiFi for the communication of the monitoring devices in an IoMT system. For example, Calcagnini et al. [42] use WiFi on a network of 45 critical medical care devices, including infusion pumps, defibrillators, lung ventilators and anesthesia machines, and show that WiFi can be effectively and securely applied for the communication of these devices. For IoT compatibility, the WiFi alliance (www.WiFi.org) proposed WiFi HaLow, which operates in spectrum below the one GHz and it is based on IEEE 802.11ah standard. WiFi HaLow meets the requirements of several categories of IoT devices, to enable a variety of use cases in industrial, agricultural, smart building and healthcare environments. WiFi HaLow enables the low power connectivity necessary for applications including sensor networks and wearables. Its range is longer than many other IoT-compatible technology options and it provides a more robust connection in indoor environments, where the ability to penetrate walls and other barriers is an important requirement. Fotouhi et al. [37] propose this protocol among others for data transmission from the coordinator within Wireless Body Area Network (WBAN) to the WiFi access points, and then from the WiFi access points towards the monitoring-control device [43,44].

##### ZigBee

ZigBee is an IEEE 802.15.4 compliant, low-cost, low-speed and low-power, wireless communication protocol. Its communication range is up to 100 m, having a data rate between 40 and 250 Kbps. It is specially crafted for Personal Area Networks (PAN) at the 915/2.4 MHz frequencies. It supports various network topologies including star, tree and mesh and it allows up to 65,000 nodes in a network [7,13,37]. ZigBee can be used in various IoT environments. It is widely used in healthcare areas for connecting sensors with the coordinator [45,46] and also for the connection among the coordinators themselves [47].

In 2009, the ZigBee Alliance introduced the ZigBee Health Care public application profile. ZigBee Health Care was “designed for use by assistive devices operating in noninvasive health care and provides an industry-wide standard for exchanging data between a variety of medical and non-medical devices” [48]. The ZigBee Health Care Profile is based on ZigBee Pro [49] and implements a fully working application layer protocol for healthcare environments. In ZigBee, The IEEE 11,073 standard protocol is supported through tunnelling.

##### WIA-PA

According to [50], WIA-PA is a Chinese industrial wireless communication standard for process automation. It has been proposed to replace standards like IEEE802.15.1, IEEE802.15.4 and IEEE802.11. WIA-PA is designed for measuring, monitoring and open loop controlling of production processes. WIA-PA is standard among other standards (wireless HArt, ISA SP100) which can satisfy the real-time and reliability and the control of process industry requirements in industrial-based systems [51]. However, Su et al. [52] propose this “industrial” protocol for medical use in a remote monitoring system as a transmission protocol in the wireless sensor network.

##### ISA 100.11a

The main goal of the ISA100 committee is to bring forward a set of standards that cover industrial-related wireless networks. In particular, these standards will address the needs of the entire manufacturing cycle, including process control, asset tracking, identification convergence of networks and long-distance applications. ISA-100.11a, the first standard of the family, proposes a mesh network topology aiming to provide secure wireless communications for process control. Its physical and data link layer are based on IEEE 802.15.4 [51]. Su et al. [52] propose the use of this protocol with the same way as they propose the previously proposed use of WIA-PA [53,54].

##### 6LoWPAN

Developed by the Internet working group of the Internet Engineering Task Force (IETF), LoWPAN is a wireless protocol with low bandwidth, limited packet size and varying address length, mainly used for allowing IoT devices to join IP networks. 6LoWPAN is its IPv6 version. The 6LoWPAN group has defined encapsulation and header compression mechanisms, which support the transmission of typical IPv6 packets over IEEE 802.15.4 based networks. Furthermore, there is a specification developed by the 6LoWPAN IETF group, the 6LowPAN over Bluetooth Low Energy (RFC 7668) [7,13].

In the case of the healthcare sector, IoMT sensors and local devices can be connected to IP networks through 6LoWPAN, allowing the interconnection among a group of sensors. Furthermore, it allows the interconnection of sensors with middleware devices or Internet-connected routers [55,56,57].

##### LoRaWAN

LoRa (Long Range) is a physical layer protocol originally developed by Semtech to support low-power and wide area networks. It uses licence-free frequencies that vary in different geographic areas (e.g., 868 MHz in Europe, 915 MHz in North America and Australia and 923 MHz in Asia). It may support long-range and low-power transmissions that may exceed 10 km in sparse areas. As LoRa defines the physical layer, there was a need for upper network layer protocols to be built on top of LoRa. LoRaWAN is a MAC layer protocol, which mainly acts as a network layer protocol. Essentially, it manages the routing and the communication between the gateways and the end devices [58]. LoRaWAN focuses on Wide Area Network (WAN) applications and is designed to enable low-power WANs with features specifically needed to support low-cost, bidirectional and secure communication in various applications like IoT, Machine-to-Machine (M2M), smart city and industrial applications. It is optimised for low-power consumption and supports large networks with millions of devices, with data rates ranging from 0.3 kbps up to 50 kbps [59].

Mdhaffar et al. [60] present an loT-based health monitoring system in which medical data collected by sensors use a LoRaWAN infrastructure to transmit the collected data to a remote analysis module in a secure way. The system mainly focuses on monitoring glucose, blood pressure and temperature of patients residing in rural areas. Since in rural areas cellular network coverage may not always guarantee an efficient data transmission, LoRaWAN is an attractive alternative. Catherwood et al. [61] present an IoMT biofluid analyser that uses LoRa and Bluetooth to support long-range data transmission. The system employs a smartphone application to create a community-based healthcare examination platform for urinary tract infections. The transmitted distance in the above prototype ranged from 1.1 to 6 Km, based on a corresponding power density reduction.

Table 2 summarises the perception and network layer protocols described above. It also provides IoMT specific examples that demonstrate the applicability of each protocol in the healthcare domain.

#### 3.1.3. Application Layer

After the medical data have been transferred by the previous layers, they are delivered to medical-specific software applications for further processing [7]. The application layer is responsible for transforming this information in a form that can be processed by the end devices and medical servers [36]. On some occasions the application layer protocols have been designed for general purpose applications [74] and may not be medical-specific. The most commonly used general purpose application layer protocols used in healthcare systems are COAP, MQTT [52] and HTTP Restful. The HTTP can be secured by applying HTTPS using the TLS (Transport Layer Security) protocol. Medical-specific application layer protocols involve medical data encoding protocols like HL7 and XML encode [75].

##### HL7

HL7 is a set of standards that allow the exchange, integration, sharing, and retrieval of electronic health information between different health entities and therefore the establishment of flexible and effective processes [76]. HL7 guarantees that the information exchanged between health systems is transparent. Its role is to define the packaging and communication details of the information exchanged between different systems. This is achieved by using a common language and by defining the data structures and data types that are required to achieve transparent and seamless integration of the interacting systems. HL7 supports not only the clinical practice but also the management, the delivery and the evaluation of health services and is recognised as the most commonly used medical-specific application layer protocol [77,78].

HL7 supports medical data standardization and enhances the collection of measured data from standard and nonstandard medical devices. The Message Exchange Standards and the Document Exchange Standards are defined for the specification of the data exchange format (HL7 v.2x, HL7 v.3, DICOM, NCPDP) [79].

##### COAP

The Constrained Application Protocol (COAP) is a web transfer protocol, specifically suited to IoT constrained nodes with limited memory and processing power. It is intended to be used in power constrained and lossy networks. COAP is under standardization by the IETF [80]. It works very well under constrained environments, e.g., in the health domain [81]. While HTTP restful communication inherently requires a significant communication and energy consumption overhead, making it unsuitable for constrained IoT devices, COAP is designed to solve these problems and enable IoT systems to use RESTful services. It is built over the User Diagramm Protocol (UDP), instead of the Transport Central Protocol (TCP), and provides a lightweight mechanism to support communication reliability. It uses the Representational State Transfer (REST) architectural style using its own protocol that is much lighter than the typical HTTP protocol. From an architectural point of view, COAP is comprised by two main sublayers: messaging and request/response [41]. COAP supports both unicast and multicast, in contrast to TCP based application layer protocols which are inherently multicast-oriented [82]. Yacchirema et al. describe a simple IoMT scenario and propose COAP as an application layer protocol for a remote monitoring system [47], in conjunction with WiFi, ZigBee and 6LowPAN at the perception and network layers.

##### MQTT

Message Queue Telemetry Transport (MQTT) was developed by IBM, with a primary goal to support lightweight M2M communications. It is an asynchronous publish/subscribe messaging protocol, operating at the application on top of the TCP stack, to allow applications/users to exchange data through networks. The goal is to provide bandwidth and power consumption efficiency, thus making it suitable for devices with constrained processing and memory resources [80,83]. By supporting a message payload from just 2 bytes (but up to 256 MB) of information, it is suitable for controlling expensive and unreliable networks [41,82] and may support lightweight communications. In the MQTT protocol, the device IDs are embedded in the payload. Separate codes are used for each operation such as for initialisation, registration, acceptance or rejection.

Dey et al. [84] create a blockchain based medical application that uses MQTT to connect various devices to an IoMT platform. Each device is identified by a unique device ID, which is encapsulated into the MQTT payload and sent to the IoMT platform. The IoMT platform is connected to the relevant blockchain using a rest API. When a device transmits the current patient status, the data is sent to the IoMT platform which in turn communicates with the blockchain using rest API. Yacchirema et al. [47] describe an IoMT scenario and propose MQTT as the application layer protocol for the communication between the cloud and an end user device like a mobile phone, in combination with WiFi, ZigBee and 6LowPAN.

##### HTTP

In several cases, the plain HTTP is used at the application layer of IoMT. Yacchirema et al. [47] describe a simple IoMT scenario that uses HTTP for the transaction between the cloud and the doctor. Dang et al. [7] use HTTP in a system with a wearable thermometer and a thermopile infrared sensor. A microcontroller board processes signals, and then the data is sent through a WiFi module to the cloud for storage via HTTP. Suciu et al. [73] use HTTP in a system that includes a wearable medical module, which is equipped with a pulse oximeter and accelerometer. This module communicates with a remote terminal unit using ZigBee. Then the data is transferred through the cloud with the help of a set of minimised HTTP requests. Sicari et al. [85] use HTTP protocol in a glycemia alarm system able to dynamically calculate the amount of insulin to be administered to patients having diabetes type 1 and type 2. Two remote programs are used to communicate via HTTP messaging: a node that implements the rules that may trigger an alarm and the end user program.

Table 3 summarises the application layer protocols that have been used in IoMT and healthcare applications, along with relevant implementations.

### 3.2. IoMT Communication Protocols in Medical Devices

The IoT communication protocols described in Section 3.1 are utilised by various medical devices that may support patients’ monitoring and treatment services [90]. We will describe the various categories of IoMT devices and we will analyse the use of the IoMT communication protocols in these devices. In Table 4 we list typical IoMT devices for the various categories discussed below.

#### 3.2.1. Physiologic Monitoring Devices

The main use of Physiologic Monitoring Devices is to monitor signals from the patient’s body and in some cases to transmit this information to other medical systems [91]. Usually, they consist of tiny wireless modules, health sensors, which gather information such as blood pressure, blood glucose, temperature, pulse oximetry or motion. These devices can be broadly divided into two categories [7,92], wearable devices (e.g., sleep apnea system) and indigestible devices. We acknowledge that other physiologic monitoring devices exist that may blur the lines between wearable and indigestible, such as scales where the user places his hands and feet and receives sensory data. For convenience, such devices are loosely classified here as “wearable” despite being of stationary type.

##### Wearable Monitoring Medical Devices

A variety of IoMT systems can be found in this category. ECG (Electrocardiography) which is used for heart monitoring and EEG (Electroencephalography) which is used for brain monitoring. Other examples of Physiologic Monitoring Devices include [93,94,95]: (a) Respiratory rate sensors which monitor patients breathing situation, (b) Activity sensors that are used to monitor actions like sleep and running, (c) Muscle activity sensors and fitness trackers that may track all fitness activities and (d) Accelerating sensors that have the ability to track the rehabilitation of the patient.

The systems mentioned above may use a plethora of IoMT communication protocols, including WiFi, Bluetooth, ZigBee and 6LoWPAN. The variety of treatment methods and of medical occasions are intentionally focused on various health monitoring WBAN-based systems. Some medical data can not be communicated directly to the gateway of the IoMT network. So, this type of data requires a coordinator to read those specific signals, convert them to data and finally send them to the IoMT network. For example, an oxygen saturation device works as follows: a finger sensor sends data to an oxymeter module which forwards data to a node for processing and data are finally collected by the IoMT network [96,97]. Body temperature and pulse sensors can receive and transmit data with Bluetooth [63,72]. A typical architecture of IoMT monitoring devices involves sensors connected directly to the human body. In custom solutions such as [72,73], Arduino modules are used to gather medical data from the sensors [12,13,64,68,69,70,98].

**Figure 2:**
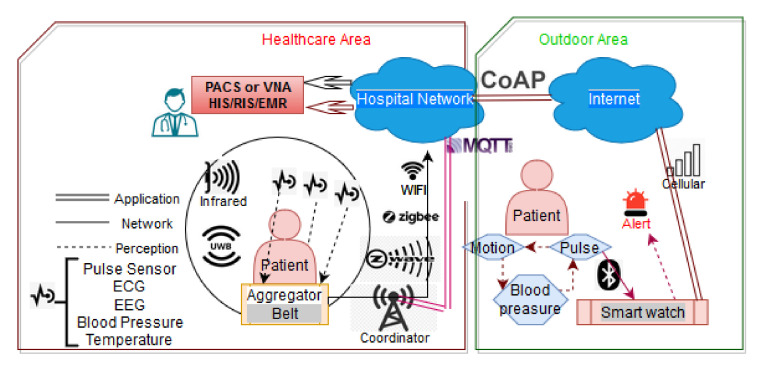
Communication among wearable sensors.

Figure 2 illustrates the communications among various monitoring devices such as wearable sensors and ingestibles. In the first case, the sensors collect information over the patient’s body at the perception layer (e.g., through infrared or UWB) and transfer it to an aggregator with the help of network layer protocols (e.g., Z-Wave, Bluetooth or ZigBee). Then, the data through a coordinator is transferred to the hospital network. Protocols like WiFi and ZigBee can be used for performing the aforementioned tasks. For certain medical applications, the information arrives through an end device to the doctor or even outside the healthcare area for remote monitoring. At the application layer, MQTT or plain HTTP can be used for these scenarios. In the second case we have a similar architecture with the previous one. In this case, a patient is located outside the hospital and wears a smart watch, which communicates via Bluetooth with the body sensors and sends the medical data via the cellular network into the hospital network.

##### Ingestible Monitoring Medical Devices

Ingestible monitoring medical devices are small, pill-like sensors. The camera pill can monitor and record the patient’s condition for a limited period of time. The ingestible devices can communicate with external systems, by sending low frequency radio waves to an external recorder [63]. In such devices, the sensors are usually connected to some gateway or aggregator, which may then be connected to the Internet, although direct Internet connection of sensors is also possible. For example, a pulse sensor is used to collect physiological signals and the sensitive temperature sensor is used for Broselow Tape (BT) measurement. The data can be sent to a mobile phone via Bluetooth [72].

**Figure 3:**
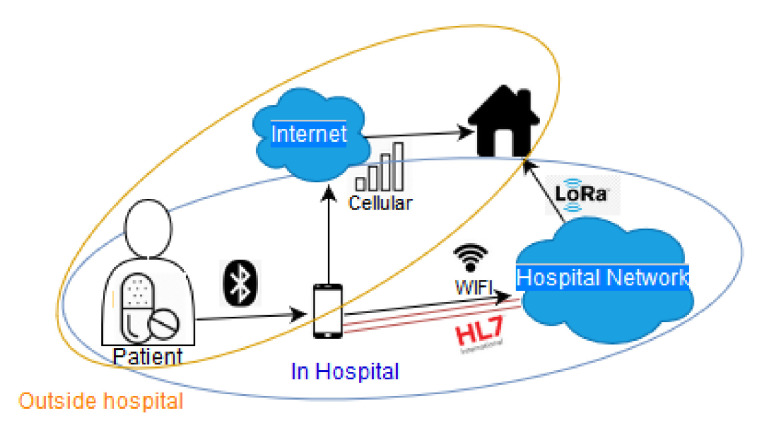
Operation of an ingestible smart pill.

Hossain et al. [45] describe an RFID based smart pill system. The system is comprised of a medicine pack sensor (an RFID chip) and sensor-coated smart pills. Figure 3 visualises the communication links in the case of IoMT camera-pills. A smart pill transfers data via Bluetooth to a mobile phone. The phone has two capabilities. First, the user of the phone can connect to the cellular network and send medical information to an area outside the hospital, like a personal doctor. Second, the phone can communicate via WiFi with the hospital network. Now the medical information is available in the local hospital network and can also be transmitted outside the hospital via a protocol (like LoRa).

#### 3.2.2. Medical Treatment Devices

Medical Treatment Devices include devices that, besides passive monitoring, are actively engaged in the patient treatment process. The most typical treatment devices are implantable medical devices and infusion pumps.

##### Implantable Medical Devices

**Figure 4:**
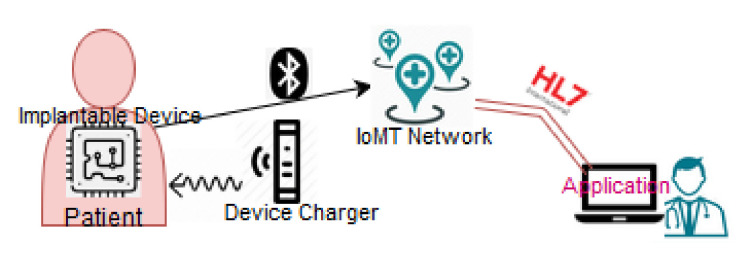
Operation of an implantable device.

Implantable Cardioverter Defibrillators (ICDs) and Cardiac Rhythmic Management (CRM) devices are typical devices of Implantable medical devices. Stachel et al. [99] describe various ICD and CRM devices where the communications are based on the NFC and RFID protocols. The authors highlight the problem when more than one patient with such a device is in proximity (e.g., same room or close to each other), since proximity may result in signal interference. Ahmadi et al. [36] describe a vaccine distribution system to enhance the supply chain management in hospitals. This system utilises various communication protocols such as ZigBee, RFID and NFC. In Figure 4 we show an implantable device that connects via BLE to the IoMT network. In this case, there exists an implantable device charger, which can extend the life of the implantable device. Thus, the patient can avoid a surgery or a painful removal procedure [100,101].

##### Infusion Pumps

**Figure 5:**
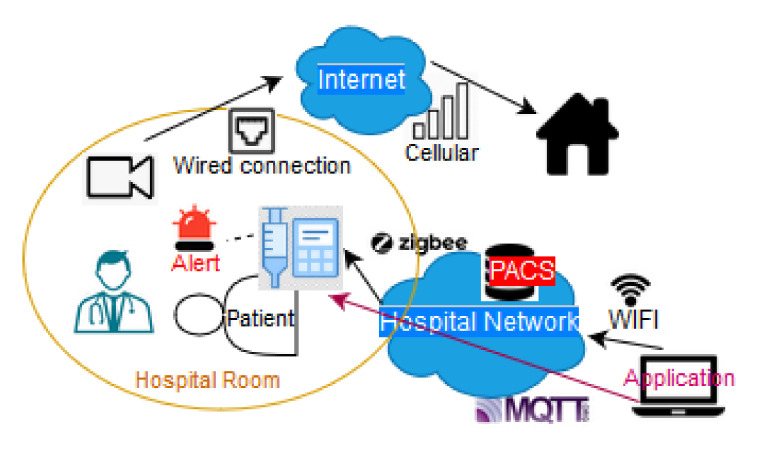
Typical communication protocols utilised for a remote treatment device (insulin pump).

Infusion pumps may be used for various treatments such as insulin and pca (Patient-controlled analgesia) pumps [102]. The protocols used by these devices are pretty much the same as the implantable and medical treatment devices. The manufacturers specify the communication protocols that the device will use depending on the module installed [103]. Several authors focus on the remote control of the pumps, which is a typical requirement in real use cases [102,104,105]. Thus, the development of authentication mechanisms is important. In some cases, patients need to have at least some limited access to their device (e.g., verifying the dosage). Full access to the device (e.g., control of the insulin’s dosage or chemotherapy drug in the body) is usually performed by doctors or medical personnel. RFID has been used for short range authentication. For remote authentication, typical public key authentication protocols, e.g., based on Diffie-Hellman have also been applied [104]. Belkouja et al. [104] describe a secure authentication system for a Medtronic insulin pump system, which is a drug infusion device, directly connected to the patient. Sicari et al. [85] present a glycemia alarm system able to dynamically calculate the amount of insulin to be administered to patients having type 1 and 2 diabetes. In Figure 5 we illustrate the procedure of remotely controlling an insulin pump. In this scenario, a patient is located in a hospital room with an infusion pump. This pump has an alert mechanism and the room has a camera monitoring system. The doctor controls the insulin pump remotely from an application through the hospital network. The doctor may also control the pump manually.

#### 3.2.3. In-Hospital Connected Medical Devices

The In-hospital Connected Medical Devices reside within the hospital environment and are either typical institutional medical devices or Surgical Robotics [3].

##### Institutional Medical Devices

Examples of Institutional medical devices include Magnetic Resonance Imaging (MRI), X-rays and Surgical Robotics. Although such devices used to be disconnected, nowadays they may be remotely controlled to support telemedicine operations. The protocols discussed here mainly refer to the application layer [106]. MRI devices use protocols and standards such as DICOM [107], HL7 over HTTP [108] or MQTT [109]. MRI is a medical imaging technique used in radiology to form images of the anatomy and the physiological processes of the body. Nabha et al. [108] describe ransomware attacks against MRI systems.

##### In-hospital Medical Robotics

**Figure 6:**
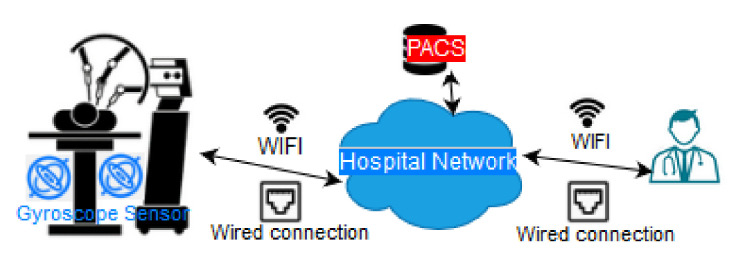
Robotic surgery-Gyroscope.

In the case of medical robotics, various categories are identified in the literature and they may include [110,111]: Surgical robotics, smart medical capsules, prosthetics, robot-assisted mental and social therapy and robotised patient monitoring systems. The communication protocols applied in Surgical Robotics include IrDA, RFID (mainly for identification), Bluetooth and HTTP at the application layer [110,111]. Bluetooth can be used for the interconnection with other smart IoT devices. For example, during a heart surgery using cardiac ablation technique the bed is equipped with gyroscope and IrDA sensors to assist the correct mapping of the human body. Information is sent from the smart clinical bed to the surgical robot to provide micrometer accuracy to the doctor. Such an example is illustrated in Figure 6. Dang et al. [7] describe a telesurgery architecture that includes microelectromechanical sensors. UWB is used to connect the sensors with the microcontroller, while a ZigBee connection supports real-time communications without delays and data losses.

#### 3.2.4. Ambient Devices

Although the ambient devices are not used for patient monitoring and treatment, they may assist these processes, e.g., managing the near-patient environmental conditions. They include patient identification devices, movement detection devices (such as gyroscopes in smart beds, motion sensors for indoor/outdoor use and vibration sensors), monitoring devices (such as IP cameras), implantable device chargers [112] and alarm devices (such as beepers).

Zanjal et al. [65] describe an RFID based system which is utilised to give a unique identification to patients and, thus, support the monitoring of patients based on their unique identification. Biometric sensors (e.g., fingerprint or eye detection) can also be used for patient identification. Other examples include gyroscope sensors, movement/motion sensors and vibration sensors which are used in the monitoring systems to alert the healthcare staff. Note that some wearable sensors are not purely medical, like the location sensors which are used to track the location of patients in cases of treatment outside the hospital. Another group of sensors are the biochemical ones and they have the ability to monitor biochemistry and detect hazardous compounds in the atmosphere [90]. The system described in Li et al. [87] also falls within this category. It refers to an IoT Healthcare Communication System that uses COAP, HL7 and other IEEE 11073 compliant protocols. Its goal is to assist the data exchange from medical and other types of devices. For message exchange, application layer protocols like HL7, DICOM and NCPDP are used to specify the data exchange format. From the sensors’ side, the IEEE 11,073 standard is used. Figure 5 and Figure 6 illustrate typical interactions of in-hospital connected devices with other treatment and ambient devices (gyroscope sensors, monitoring and alarm devices).

#### 3.2.5. Other ICT Devices

These are not IoMT devices but other ICT (Information and Communication Technologies) devices that reside within the healthcare environment and may interact with the IoMT devices. We list them for completeness. In the healthcare environment such systems may be medical servers, coordinators, databases with health data, real-time monitors, mobile devices, laptops and network equipment [12,75,113].

#### 3.2.6. Monitoring and Handling

All devices connected to the IoMT network to serve medical purposes belong in the monitoring and handling group. Any medical information obtained from any medical sensor node is intended to pass through the IoMT network and reach a physical person (such as a doctor). These devices, combined with running protocols and software (EL7), are responsible to provide information in a form readily recognisable by the natural person. These devices, on many occasions, can control and handle medical devices which are connected to the patient. A tablet, a mobile phone or a personal computer may belong in this category [47,65].

#### 3.2.7. Coordinators

The coordinators are devices between the sensor and gateway. A coordinator node acts as a relay node that collects the data and transmits it to the primary medical device for example (a cell-phone application) or a small wireless device, for example (an access point connected to the Internet) [114]. They are usually close to the human body and they receive radio waves from specific sensors and transmit the data to the gateway [7]. Some sensors, like a temperature sensor, can transact with the coordinator via infrared. The coordinators most often use protocols like Bluetooth or ZigBee.

## 4. Security in IoMT Communication Protocols

In this section we will examine the security status of these IoT communication protocols that are specifically used in IoMT devices, as identified in Section 3. First, we briefly present the security features that exist in each of the above protocols. Then, we discuss current protocol security weaknesses and present potential attacks that, based on the literature, are feasible in each protocol implementation, along with brief technical aspects.

### 4.1. IoMT Protocol Embedded Security Features

We briefly describe the prominent security controls that were taken into consideration during the design and development phase of the most common protocols used in IoMT. For reasons of consistency, our analysis will follow the IoMT protocol taxonomy presented in Section 3.1.

#### 4.1.1. Perception Layer Security Issues

##### Infrared

Infrared (IR) communications have no embedded security controls. Anyone that can intercept the IR beam can read data sent between the transmitter and the receiver. Since the IR technologies are directed beams and only work in very close proximity, security threats were considered out of the scope of their threat model; for example, the attacker has to be very close to the IR device and be equipped with the appropriate material. Recent attacks, however, demonstrate the feasibility of such threats [3].

##### RFID

In RFID communications the embedded data are unprotected and read only. By default, RFID implements no protection or authentication controls against tag scanning, both for the tag itself and the scanner. This paves the way for integrity attacks on the tag data, unauthorised cloning of tags, confidentiality attacks against devices, equipment or medical data, unauthorised tag tracking, replay attacks and DoS attacks [115,116]. Still, RFID tags usually hold some security mechanisms, mainly for symmetric key encryption. Some other implementations utilise some sort of signature embedded information on RFID tags to promote integrity control and tag authentication.

##### NFC

Security protocols for NFC are standardised in ECMA-385 and ECMA-386. ECMA-385 defines shared secret (SSE) and shared channel (SCH) security services for defending against eavesdropping and data integrity attacks. SSE implements the protocols for key exchange and key derivation and confirmation, while SCH is used for data encryption and data integrity checks; both using the Advanced Encryption Standard (AES) algorithm with 128-bit key. ECMA-386 defines the cryptographic mechanisms to be implemented in these services [117]. NFC utilises three modes of operation: Read/Write, Peer-to-Peer and Card Emulation Mode. NFC uses smart cards and their underlying secure elements. Each mode utilises different protocols and thus is prone to different security vulnerabilities.

##### Bluetooth/BLE

After the major update of 2007, Bluetooth can operate under four (4) different security modes. The strongest, mode 4, uses secure simple pairing (SSP) for service-level security. The bluetooth device authentication is performed before connection establishment and utilises stream cipher encryption, which reduces potential Man-In-The-Middle (MITM) attacks. Its encryption algorithm utilises the master device’s address, clock time, and a key to establish symmetric encryption between devices [118]. The Bluetooth device chips are assigned unique identifiers.

##### Z-Wave

Z-Wave utilises encryption, behaviour detection and proximity security mechanisms. The protocol also protects the confidentiality, source integrity and data integrity of its data through the “Security” command class. Payloads and frames are both encrypted and integrity protected through AES encryption using three shared keys [119].

##### UWB

UWB generally follows the security requirements as defined in IEEE 802.15.4. In addition, as UWB offers spatial awareness or node positioning information to devices, positioning attacks are of special concern. According to the FiRa Consortium, a security extension is added, as specified in IEEE 802.15.4z and introduces secure ranging schemes to provide mechanisms for defining device positioning and distances measured between devices in a secure and protected manner for both high/low pulse repetition frequency devices (LRP/HRP). Another embedded characteristic that can be used to mitigate some positioning attacks is the size of the UWB symbol (a form of distortion of a signal in which one symbol interferes with subsequent symbols) because the measured distance cannot decrease below the symbol size [120].

#### 4.1.2. Network Layer Security Issues

##### WiFi

Wireless security (WiFi) strictly follows the IEEE 802.1X Standard authentication mechanisms for devices wishing to connect to wireless networks. Current WiFi systems security implements the WiFi Protected Access 2 (WPA2) standard, which encrypts data sent over wireless networks with a 256-bit key. Wireless technologies usually implement a wide range of security measures, although these are not enforced by default in all networks. Besides the WPA2 and IEEE 802.1X security specifications, other common measures for WiFi include SSID hiding for covering the service identifier and partially protecting against scanning, MAC filtering and static IP addressing for device authentication. Nevertheless, these security features are not considered robust enough to thwart potential attacks.

##### ZigBee

ZigBee has adopted various security controls. ZigBee implements services for key management and distribution along with controls on network and packet frame security. Security controls are implemented at the application layer [49]. Since ZigBee is based on the “open trust” model, underlying protocol stack layers trust each other and cryptography is implemented only between end-point devices [121]. ZigBee uses 128-bit AES encryption and devices must preshare keys in order to be able to securely communicate [122]. For packet frame security, ZigBee utilises frame-protection mechanisms at the network layer both for outgoing and incoming packets [49]. At the network layer security, ZigBee has a network key. It is an essential key used for encryption between the nodes within the same network. It is randomly generated by the trust centre and it is also preconfigured by a link key in the application layer [123].

At the application layer, ZigBee uses a global link key and a unique link key [124]. The global link key is used between the trust center node and a normal node; it is also preconfigured and allows the manufactured nodes to join the network. The unique link key is optional and used for the communication of a pair of nodes. It is also one of the three keys below (The preconfigured unique link key, the trust centre link key and the application link key).

##### WIA-PA

A WIA-PA architecture for device authentication is proposed by Wang et al. [125]. WIA-PA uses a join key shared between a device and a security manager to authorise access. Besides these, WIA-PA has no other default security controls applicable on relevant networks.

##### ISA 100.11a

ISA 100.11a supports various security mechanisms to achieve message authentication, data confidentiality and protection against replay attacks. For device authentication a linchpin is used to prevent a spoofed device from joining a network. Data confidentiality and message integrity are based on the AES 128 encryption. Finally, replay attacks are thwarted by applying freshness checks on the communication messages. The underlying security mechanism for message freshness checks is based on accepting messages received within certain time frames after their transmission [126].

##### 6LowPAN

6LowPAN is a combination of the IEEE 802.15.4 standard and the IPv6 protocol. At the link layer, the IEEE 802.15.4 includes encryption and authentication security features but, as of 2012, it does not specify keys management protocols and policies [127]. 6LowPAN implements seven security modes, all established around the AES cipher suite. At the network layer, 6LowPAN implements the security mechanisms detected in IPv6. IPSec is included and provides end-to-end security at the network layer, namely Authentication Headers and Encapsulated Security Payloads (ESP). IKEv2 is often used as the key management protocol associated to IPSec [128]. At the network layer, 6LowPAN utilises the Datagram TLS (DTLS) protocol based on TLS to secure network traffic.

##### LoRaWAN

LoRaWAN’s architecture uses two layers of cryptography. First, it applies a unique network session key (NetSKey) that is shared between the network server and the end nodes. Second, it uses a different application session key (AppSKey) for end-to-end encryption at the application level. The AES encryption algorithm is used to support authentication and data integrity for packets exchanged through the LoRaWAN network. Keys are set to the typical, for AES, 128-bit length.

Thus, LoRaWAN relies on symmetric cryptography, which requires underlying mechanisms for secure key sharing. To support this, the LoRa Alliance [129,130] provides back end interfaces that isolate the storage of root keys in the join server; the idea is that the join server becomes a trusted party, regardless of the underlying network. The LoRa Alliance also provides secure element solutions for hardware-based physical protection against tampering. For ensuring device and network security, typical trust management techniques must be employed, including the use of trusted (certified) service providers and the use of public key certificates in order to certify the devices used.

#### 4.1.3. Application Layer Security Issues

##### HL7

HL7 focuses on application level data transmission and not on security itself. Its goal is to provide a common ground for transmitting data between systems, through international standards that describe data sharing between various healthcare providers. Thus, it lacks any built-in security controls. Authentication, message encryption or message integrity controls are not inherently supported by HL7. HL7 implementations usually rely on security features provided by the underlying communication protocols, if such security features are available.

##### HTTP

The HTTP protocol includes two types of authentication mechanisms, the Basic and the Digest authentication. The basic authentication sends credentials without any encryption, while in the Digest authentication the header will contain the digest value together with a nonce (a number that is used only once) and a URL path (realm) [131]. Both methods are not considered secure without the use of SSL/TLS encryption.

##### COAP

COAP utilises four modes of operation, each with its own security characteristics. The NoSec mode implements no security controls. The SharedKey mode uses a preshared key for all communicating parties, while the MultiKey mode utilises different, unique keys for each device participating in the network. Finally, in the Certificate mode, COAP provides end-to-end security through the use of certificates together with the aforementioned shared or multi key mechanisms [132]. Additionally, COAP uses multicast messages to manipulate resources at different groups of devices at the same time. COAP does not contain any embedded authorization mechanisms [81].

##### MQTT

MQTT does not impose any security mechanisms by default. It is designed to operate in already secure networks, for example within restricted, local environments [81]. Still, MQTT does implement a four-way handshake mechanism to ensure message delivery and service availability [80].

### 4.2. IoMT Protocols Security Weaknesses and Attacks

Since IoT protocols are mainly designed to be energy efficient and to efficiently handle node connectivity in complex ad-hoc environments, security comes as a secondary characteristic. This, coupled with the fact that most IoMT applications often rely on diverse technologies with different embedded properties, each of which often has its own set of security issues [116], eventually led to many widespread IoT protocols missing basic security mechanisms. This became painfully obvious in September 2016, when the Mirai botnet crippled multiple services by exploiting IoT devices like home routers, monitors and surveillance cameras [133]. Other similar attacks closely followed, like the massive Distributed Denial-of-Service (DDoS) attack on Dyn in October 2016 [134].

This section presents the most prominent security weaknesses, as recorded in protocols used widely in IoMT. The presented vulnerability landscape is structured based on the architectural layers used in IoMT, namely the Perception, the Network and the Application layer.

#### 4.2.1. Perception Layer Weaknesses and Attacks

##### Infrared

IrDA supports point-to-point connections and requires direct line-of-sight communication between two devices with infrared sensors. The Infrared technology lacks technical support even for the most basic of security measures. Despite its very small range, it is possible for attackers to eavesdrop on data transmitted by intercepting the reflected infrared-light and filtering out the surrounding ambient noise [135].

##### RFID

In RFID, confidentiality attacks can be performed on the physical and network layer. Attacks mostly raise confidentiality issues on shared data and location privacy concerns. Attackers can often compromise sensitive health data related to treatments stored in tag data [136]. Even solutions such as encrypted RFID implementations are known to be susceptible to side channel attacks due to their passive nature [137]. Both the active (continuously transmitting) and the passive (electromagnetic field) RFID systems are known to have several weaknesses. For example, researchers have shown that intended interference may cause RFID systems to fail and directly impact the physical safety of a patient, e.g., by switching equipment off or by inducing service disruptions [138].

##### NFC

Near Field Communication (NFC) regulates radio frequencies to allow for data exchange between two devices in close proximity. The NFC standard provides no stringent security measures against proximity attacks. Typical Man-In-the-Middle (MITM) attacks using simple antennas can cause breaches of data confidentiality or corrupt signals, resulting in integrity or Denial-of-Service attacks.

Other NFC MITM attack variations involve the Proximity Inductive Coupling Card (PICC). The PICC is practically a transponder that may be read or written by a proximity reader based on the ISO14443 standard. Such tags do not have any power supply. They are powered by the electromagnetic field of the reader PICC attacks exist that utilise the proximity coupling device (PCD) and exploit its protocol challenge-response requests using a malicious NFC reader and an emulated PICC. However, these attacks are prone to timing restrictions [139].

NFC is also vulnerable to DoS and partially to data modification attacks, either through signal corruption or through bit manipulation in specific types of NFC card modulation. According to [140], partial bit modification is applicable for the modified Miller encoding that utilises 100% amplitude shift keying (ASK). The same attack is also feasible in the Manchester coding with 10% ASK.

##### Bluetooth/BLE

By default, Bluetooth encryption only encrypts the payload and not the entire packet. Many medical devices implement the same interface type and specific channels for similar services, such as for device model verification and service listing. This can be exploited by attackers to get information on existing vulnerable vectors [141]. Each Bluetooth device chip is assigned a unique identifier. Still, there exist methods able to bypass this restriction and alter multiple device information. Device addresses on certain chips can be modified through firmware modification (e.g., using the bdaddr app). Device name and class can be modified through software injection with the use of the Hciconfig software.

Another attack involves matching the Bluetooth connection’s frequency hops and then capturing data in that frequency range. Such attacks can sniff and capture Bluetooth packets for MITM confidentiality attacks [142]. Although current implementations of the protocol utilise some security controls against MITM (as mentioned earlier), still researchers have shown that weaknesses do exist. For example, a BT-SSP-Printer-MITM attack on the “Just Works” feature connection of Bluetooth has allowed attackers to pose as both the user and a printer. This attack performs a DoS that forces legitimate users to reset Bluetooth associations between devices [143].

Other MITM attacks utilise a weakness in the Bluetooth address verification. These reflection attacks mimic the BD_ADDR of target devices to bypass authentication, although such attacks do not completely bypass the encryption mechanisms [144].

Other known Bluetooth attacks can: (i) exploit the potential to brute-force Bluetooth PINs from pairing process packets, (ii) jam signals and create DoS on services and (iii) send unsolicited messages to enabled devices (BlueJacking attacks). DoS attacks vary in type and technique, ranging from device duplication attacks, Piconet spam to prohibit legitimate device connection and Negative Acknowledgement attacks to slow down connection through endless loops have also been related. Still, most efficient Bluetooth attacks recorded tend to exploit vulnerabilities in specific device instances, rather than exploit elements of the Bluetooth protocol itself [144].

##### Z-Wave

The Z-Wave protocol does not enforce a standard key exchange protocol. Custom key establishment protocols are known to be exploited by attackers. In 2013, researchers have found a Z-Wave implementation that could be hacked by exploiting the custom key establishment protocol and an implementation feature that allows multiple protocol executions for a single device [145]. Z-Wave S2 tries to alleviate such issues by implementing standard Diffie-Helmann key exchange protocols.

Other attacks on ZWave-enabled devices include impersonation attacks and node spoofing attacks to bypass network checks, as well as BlackHole attacks. Impersonation attacks are able to fake device sources by spoofing the frames originating from the controller or from another device. This is achieved because Z-Wave devices implicitly trust the source and the destination fields of the MPDU (MAC protocol data unit) aggregation frame: a handy fact when trying to bypass device authentication checks [119]. Black Hole attacks involve intermediary nodes that “silently drop application frames when it is expected to forward them” [119]. Still, Z-Wave networks are vulnerable to such attacks only if malicious nodes exist and are assigned by the controller to be part of a path between two devices.

##### UWB

Since UWB is a distance-based protocol, it is vulnerable to physical layer attacks, such as the early detection and late commit (ED/LC) attack described in Singh et al. [146].

In the “Same-Nonce” attack [147,148], various events such as wrong access control configuration or a power failure may result in clearing the Access Control List and then sharing the same nonce and the same security key for two consecutive messages. An eavesdropper can recover partial information by XOR-ing these two consecutive cipher texts.

#### 4.2.2. Network Layer Weaknesses and Attacks

##### WiFi

The most common security weaknesses of the WiFi protocol are the lack of granular device authentication, the limited protection of service integrity besides encryption and the protocol’s structural weakness against denial of service attacks on the wireless network an signal itself. Specifically for denial-of-service attacks, these can be directed to different layers of a WiFi implementation. Some attacks focus on the physical layer (e.g., rogue stations, node tampering, proximity attacks and WiFi Channel Collision), some on the software layer (race conditions, packet replay attacks or battery exhaustion [116]) and some on the network layer (network flooding, wormhole attacks, etc.).

Common attacks on medical WiFi networks also include peer-to-peer and eavesdropping attacks, since connected devices are vulnerable to other devices connected to the same network. In addition, WiFi networks can be exploited through MAC spoofing, where a malicious device spoofs the MAC address of an existing medical device. This way, the malicious devices can launch integrity and confidentiality attacks against all data travelling to the spoofed device.

##### ZigBee

ZigBee allows key reuse among layers of the same device and uses the same security level for all devices on a given network and all layers of a device [124]. ZigBee’s exploitation can be broken down into two discrete categories: implementation and protocol vulnerabilities. Implementation vulnerabilities mostly focus on encryption configurations, such as utilising insecure key transportation for preshared keys, reusing Initialization Vectors (IVs) during encryption and vendors installing default link keys for all the devices or sending security headers in clear text on auxiliary frames [149]. Still, all the aforementioned vulnerabilities are instances of ZigBee implementations and are not protocol specific vulnerabilities.

ZigBee’s protocol vulnerabilities mostly relate to vulnerabilities inherited from 802.15.4. Acknowledgement packets (ACKs) have no integrity checks, only sequence numbers that can easily be intercepted. Attackers can forge ACKs at the MAC layer with multiple adverse effects, e.g., disassociate services and access control from legitimate devices. Other attacks on the MAC layer involve flooding that causes DoS [150].

Concerning different layers, other ZigBee vulnerabilities include the insufficient registration of network keys for encrypted communication between devices. Network key updates encrypt new keys with old network keys before broadcasting them to network devices. This does not preserve forward secrecy. Opting out of these updates is even worse, since it will eventually allow reuses of Initiation Vectors which may lead to key compromise [150].

Another detected ZigBee vulnerability exploits the lack of verification in devices’ PAN IDs. This allows attackers to reset to Factory defaults all device network connections. This in turn paves the way to hijack device networks through crafted Beacon messages and associate the devices to malicious networks [151]. Other ZigBee attacks exploit energy-consuming services to deplete power, especially on portable devices [152].

##### WIA-PA

WIA-PA is a standard that implements security controls against attacks such as eavesdropping and Traffic Analysis. Nevertheless, WIA-PA still has some vulnerabilities. Specifically:

- It does not provide any nonrepudiation security service as it lacks a public key encryption algorithm.
- WIA-PA can defend against most Sybil attacks. However, attackers can disguise as a proxy node and send join responses to devices trying to join the network during the nonencrypted join request phase. This makes the network vulnerable to Sybil-type attacks [153].
- Since the primary stage of secure authentication, the request is not encrypted. This security flaw can make the WIA-PA network vulnerable to DOS and Sybil attacks.
- The security management framework and its interface are not determined in detail.

Dos attacks are applicable on WIA-PA during nonencrypted join requests [153]. The attacker can recalculate the Cyclic Redundancy Check (CRC) and keep sending malformed packets. This renders the receiver very busy because it has to perform integrity checks each time a packet is sent.

In a WIA-PA network, a wormhole attack is easy to be launched by an adapter and wired devices connected within the WIA-PA network. If an attacker has the masters key information, he can establish a connection between the two routing devices using wireless tunnels which can simply have stronger receiving and sending power.

Jamming attacks can take place when the attacker can control the hopping sequence and manipulate network data transmission to consume node energy [125].

##### ISA 100.11a

ISA100.11a defines security functions for use over IEEE 802.15.4 wireless networks. Its network and transport layers piggyback on 6LoWPAN, IPv6 and UDP [54]. As such, the ISA 100.11a transport layer provides end-to-end encryption. Potential security issues stem from inherent vulnerabilities of the aofrementioned technologies. In addition, in ISA 100.11a, security has some optional characteristics. New devices get a join encryption key that is used to authenticate the device on the network. The security manager will provide communication keys after device authentication. Still, the use of a join key is optional in ISA100.11a. Implementations can instead use a global key with no security guarantees to join a network. Last, encrypting messages in ISA100.11a is optional and not mandatory [54].

Optional characteristics pave the way for numerous attacks. A replay attack can take place if the system lacks message authentication and freshness of communication messages mechanisms. In addition, sniffing and spoofing attacks along with data falsification are feasible if systems lack message encryption [154].

Another important fact is that the ISA protocol usually connects devices with special characteristics that may be exploited by attackers [155]:

- Devices have limited storage space, computing capability, bandwidth and communication capability that may be exploited for attackers to launch DoS attacks.
- Devices have limited power supply that can be a target of power depletion attacks.
- The dynamic nature of its network environment can affect the network performance.

##### 6LowPAN

Since 6LowPAN utilises the IEEE 802.15.4 standard and the IPv6 routing protocol; its security considerations are tied to these technologies. The 6LowPAN attacks focus either on the IP network or on the radio signal. One class of attacks involves the use of malicious intermediary network nodes that attack a 6LowPAN network from the inside. Such attacks involve signal jamming, replay attacks to cause address depletion and flooding attacks to cause DoS against legitimate devices. Generally, 6LowPAN is affected by most of the attacks known to exploit 802.15.14 protocols [156].

Another 6LowPAN vulnerability was exploited in [157] under the assumption that a node’s IP address remains unchanged. Using traffic analysis, authors correlated unrelated network traffic using 6LowPAN fixed addresses and managed to infer private information.

6LowPAN also suffers from vulnerabilities at its fragmentation mechanism. Authors in [158] identified two design-level vulnerabilities that enable attackers to selectively prevent correct packet reassembly on the target nodes using a single protocol fragment.

##### LoRaWAN

Several vulnerabilities have been detected in LoRaWAN that lead to multiple types of attacks. Replay attacks were found to selectively cause DoS on specific devices. Exploits exist that managed to allow plaintext recovery of passwords and malicious message modification, opt to deny delivery reports and even cause battery exhaustion on connected devices.

Plain text recovery attacks involve resetting frame counters without rekeying devices. Denial of packet delivery attacks used the caching and replaying of ACK packets. The battery exhaustion attacks transmitted falsified gateway beacons to repeatedly wake up sensors. Selective DoS attacks utilised a dictionary of past messages to inject old messages in counter gap windows, while MITM attacks allowed attackers to modify network content and cause integrity breaches if an attacker’s device has network access between the devices and the LoRaWAN App Server [159].

#### 4.2.3. Application Layer Weaknesses and Attacks

##### HL7

HL7 was built insecurely. Since HL7 implements no stringent security measures, HL7 security flaws relate to implementation issues. Ad-hoc security controls deployed in various implementations have often been found to have various encryption and authentication vulnerabilities. Such implementation-specific vulnerabilities pose a significant threat to hospitals and patients since they may render personal and sensitive patient information susceptible to cyber attacks, data privacy breaches, or even worse, harming patients.

For example, an attacker could modify many lab results to read from “normal” to “severely ill”. Some of the most major vulnerabilities detected stem from the lack of encryption between devices due to insecure configuration [160]. Often, message sources are not validated by default and do not utilise a proper two-way authentication protocol. This can lead to device spoofing or integrity attacks on communications. In addition, the size of HL7 messages is often not validated. This can lead to DoS and flooding attacks on receiving devices.

Another type of attack on networks using HL7 involves the lack of authentication that allows rogue devices to disrupt functionality through malformed ACK messages. As far as integrity attacks are concerned, the modest payload size and the lack of integrity checks provide attackers with the opportunity to fuzzy input and potentially change vendor, device, message version and fields between communicating devices [160].

##### HTTP

HTTP is insecure by default and is therefore exposed to typical attacks like eavesdropping, injection and manipulation attacks. Default HTTP implementations are not encrypted. Encrypted HTTP (HTTPS) is used for end-to-end encryption. Below, we present some of the most common threats applicable in HTTP protocol communications. As with HL7, existing vulnerabilities and potential attacks relate to implementation issues and not on the HTTP protocol itself.

- Waste Flood: An attacker tries to open a connection to a known HTTP port like 80 or 443 to send excessive binary data using the the http protocol.
- GET Flood: The attacker uses the HTTP GET request method on a large scale to overload servers and cause DoS on provided services.
- HTTP methods flood: Attacks use the HEAD and POST methods in conjunction with a GET flood attack to overload the server code.
- HTTP fuzzer and faulty fields: The main purpose of these attacks is to break code execution at the server by sending junk data or bad values inside specific HTTP protocol fields. For example the attacker can send a G(3)T request (instead of a GET request), or the attacker can send traffic to version HTTP 1(,)1 (instead of HTTP 1.1).
- Low and slow rate attack: In this case an attacker sends illegitimate HTTP traffic at a very low rate, in an effort to avoid being perceived by intrusion detection mechanisms [161]. In IoT, such attacks often try to deplete the energy of devices.

##### COAP

COAP works in conjunction with the Datagram Transport Layer Security (DTLS) which is the official security protocol to support security in COAP. DTLS comes with its own security limitations, such as large messages and handshake compression, and does not suit the COAP proxy modes [162]. In [132], the authors indicated an end-to-end communication issue for some scenarios that require resource access from COAP back-end servers. The issue arises from proxies having to decide if the DTLS implementation will be multicast or unicast message. Although security mechanisms have been instigated in COAP [163], still the protocol does suffer from common attacks like:

- Parsing attacks, where a node could execute malicious code.
- Cache attacks, where a proxy server can gain control of a part of the network.
- Amplification attacks, where an attacker can use end devices to convert a small packet into a larger packet and launch DoS attacks. COAP Servers can mitigate amplification attacks by using Blocking/Slicing modes.
- Cross-Protocol attacks, where packet translation from TCP to UDP is liable to attacks.
- Spoofing attacks.

##### MQTT

MQTT has several security mechanisms for data encryption and authentication [164], which however are not provided and/or set up by default. MQTT protocol security is based on an authentication mechanism without encryption capabilities [165]. Attackers can perform traffic analysis to extract valuable information from data in-transit. Sniffing attacks mostly target information related to the IP broker (usually public IP address), Data payload and Port number of devices. Below we compiled a list of threats that can accrue from the security gaps of the MQTT protocol [164].

- Data Privacy: By default, MQTT does not provide any embedded data encryption mechanism. Whether the broker device (the master device that handles the traffic in the MQTT protocol) uses authentication mechanism or not, the intruder can still sniff the data when transmitted between the broker and a simple IoT device node.
- Authentication: If the attacker is in the same network with the publisher, he can sniff traffic and wait for a “Connect” packet from the publisher. Such packets can contain the username and password used to connect to the broker. Additionally, attackers can monitor for the “Keep Alive” packet. During the authentication process, there is a header in this packet which indicates how long the IoT device will remain connected to the broker. Therefore, when time expires, the device will resend the “Connect” packet to restart the connection.
- Data integrity: Intruders can modify data during transport between IoT nodes. They can use a compiled filter to modify the packages after the successful execution of ARP poisoning to reroute network connections to pass through the intruder’s computer (Man-In-the-Middle attack).
- Botnet Over MQTT: In this case, the attacker uses the Shodan search engine to find a device with the role of a broker. Then, he tries to transform it to a free broker server that connects himself to the victim’s device. In this scenario, the intruder uses the “unsecured” broker as an arbiter to create an IoT botnet.

### 4.3. Proposed Mitigations to Common Protocol Weaknesses

Mitigating attacks on the IoMT networks rely heavily on the peculiarities and distinct characteristics of each implementation instance. Each installation has its own configurations and custom implementations of protocols and devices, which makes its attack surfaces unique. Still, most IoMT networks share some characteristics depending on the technologies used. Each implemented technology and protocol can be coupled with mitigation measures, based on the weaknesses of that technology and the body of knowledge concerning its recorded attacks.

In this subsection, we briefly present current trends and the most documented mitigation measures for securing modern networks and protocol implementations in protecting the supply chain in the IoMT for medical infrastructures and services.

#### 4.3.1. Perception Layer Mitigations

##### Infrared

Since attacks against infrared communications require a line-on-sight interaction with the target, physical security controls can be applied to protect eavesdropping or jamming attacks.

##### RFID

RFID is a technology used in devices with very low-power features which makes common protection mechanisms difficult to implement. However, interesting custom authentication mechanisms have been proposed by researchers. Song et al. [166] propose a strong authentication protocol for RFID tags to assure tag location privacy, replay attack and Denial of Service attack protection, as well as backward and forward traceability protection. Sun et al. [167] propose a hash-based RFID security protocol that provides forward privacy. The goal of this protocol is to protect the RF tag from tracking attacks, by observing previous unsuccessful sessions of the tag.

Cvitic et al. [168] identify various limitations and propose partial solutions. They propose a lightweight encryption function using Identity Based Encryption, called VLFSR. It is efficient and effective against eavesdropping, location tracking, replay attacks, MITM and desynchronization attacks. To protect from tag cloning, they propose the use of synchronised secrets that can detect cloning attacks and pinpoint the different tags with the same ID. For eavesdropping and replay attack protection, they use dynamic password and custom system authentication systems.

##### NFC

In theory, NFC implementations suffer from MITM attacks, but real-world executions of these attacks are very difficult to deploy due to NFC’s architecture and distance limitations [169]. Eun et al. [169] propose two security services to cope with such attacks: the SSE (Shared SEcret service) and the SCH (Secure CHannel service). SSE generates a secret key for secure communication between NFC devices, while in the meantime, key agreement and key confirmation are taking place. A key generated through the SSE service is used so that the SCH service provides the communication between NFC devices with confidentiality and integrity.

Madlmayr et al. [170] presented a list of known security issues of the NFC protocol, specifically:

- Denial of Service and phishing attacks.
- Issues due to the fixed unique ID and in-device security.
- Transactions over the peer link and during the relay of data transferred over the RF.

Additionally, they proposed some practical measures for each of the aforementioned attacks, and specifically:

- Create a button to turn on the NFC device, with a user able to operate this device consciously.
- Only random NFC ID should be used for anti-conflict (without the use of ID-based systems).
- Allow the NFC admin to use the security features of a secure peer-to-peer NFC connection.
- Create a whitelist with authenticated and signed applications.
- Use of signed tags against phishing attacks.

For the NFC controller component, James et al. [171] proposed a Single and Multiple antenna design to mitigate attacks like denial of service on devices, data corruption, tag cloning and low battery. In addition, authors proposed encryption controls to prevent unauthorised access or data corruption. SSL, VPN technology and encryption can mitigate most of the attacks (e.g., Sniffing, DoS, Eavesdropping and Data corruption). Threats like DoS, Data encryption and Unauthenticated access can be avoided by encryption.

For the eavesdropping attack Haselsteiner et al. [140] established a secure channel with a standard key agreement protocol based on Diffie-Helman, RSA and eliptic curves. For addressing data corruption, authors proposed to check the RF field of NFC readers during data transmission. For data modification, the provided solution proposes a secure channel where NFC devices check the RF field while sending data. For data insertion, the proposed solution involves having devices answer to requests with minimal delays through secure channels. Finally, for MITM attacks, authors propose the use of active-passive communication to continuously generate RF field data using one of the valid communication parties.

##### Bluetooth/BLE

Published research argues that devices connected through BLE may be vulnerable to numerous threats on all communication layers. Still, BLE implementation utilise multiple security controls to mitigate attacks. Some solutions entail the use of AES-CCM encryption to achieve confidentiality and integrity. The data channel packet data units (PDUs) can also be authenticated with a 4-byte MIC module [172]. To secure all data, including also the metadata, an innovative approach is based on the black network concept proposed by Chakrabarty et al. [173].

Other solutions use an AES coprocessor for encryption and decryption purposes and a separate Bluetooth module [8,174].

A frequency hopping mechanism that reduces the risk of eavesdropping on transmitted packets can be used, since frequency hopping is more relaxed in BLE [7].

Lonzetta et al. [175] propose a group of practical, technical and application based solutions to protect BLE against attacks. Specifically, they mention the following practical solutions:

- Device settings and BLE version should be constantly kept updated.
- Update authentication credentials.
- Pair only with authenticated devices and avoid auto-pairing.
- Turn off Bluetooth modules when not necessary and set device to undiscoverable mode.
- Utilise strict pairing policies.

In the case of technical solutions, they emphasize the following:

- Use of combination keys instead of link or unit keys to prevent MITM attacks.
- Use link encryption to prevent eavesdropping.
- Do not use multihope communication when encryption is not supported.
- Use of encrypting broadcast interceptions and use of security mode three implemented at the link layer with a 128 bit encryption key.

##### Z-Wave

Z-Wave offers confidentiality, source integrity and data integrity services through AES (mostly 128) encryption, policy driven and behaviour detection mechanisms. Z-wave includes a security command class where the application frames may be encapsulated in a security frame that is both encrypted and signed. The frame is further secured based on symmetric encryption using AES with three shared keys, known by every node of the network requiring the security service [119]. However, there are some techniques which help to further protect IoT devices using Z-wave [176]:

- Hide the WLAN SSID: Since an attacker should be able to use a passive network scan tool to locate the WLAN, the administrator can hide the Service Set Identifier (SSID).
- WPA2 should be used instead of WEP. A WEP 128-bit key can be cracked in just few minutes with free available tools.
- MAC address filtering allows the WLAN administrator to choose a number of devices that are authentic and should be acceptable in the network. This procedure must be combined with WPA2 or AES-128 bit encryption. Otherwise, a spoofing attack can take place.
- Use a Reverse Proxy Server.
- Inspect log files mostly for prevention or direct intervention.

##### UWB

UWB is a distance protocol that is menaced by attacks that differentiate the distances between the nodes. A Verifiable Multilateration (VM) algorithm is proposed in [177] that applies verification triangles to detect a distance enlargement attack. IN [178], authors propose a mobility-assisted secure localization scheme (SLS) to deal with external attackers who intend to manipulate the distance measurements. Other papers also present a location-based secure authentication scheme to prevent external attacks [120,179].

Other literature proposes that UWB is prone to the same “Single-nonce” attack detected on Zigbee [147]. Illicit and/or intended power failures of an IoT network can make UWB networks vulnerable to a Same-Nonce attack that results in a clear Access Control List. According to literature, a simple and practical countermeasure is to store the nonce states in a Non-Volatile Memory (NVM) and recover them after each power failure.

There also exists a modulation technique that enhances the extended mode of 802.15.4f with cryptographic mechanisms at pulse level. This aims to mitigate physical-layer attacks that exploit node distancing, while retaining the range and performance of the extended mode. According to Singh et al. [146], UWB with pulse reordering (UWB-PR) is the first modulation technique to prevent ED/LC attacks independently of the communication range offered.

#### 4.3.2. Network Layer Mitigations

##### WiFi

An IoT system/network that communicates with WiFi has some technical design characteristics [180].

- WiFi AP access points and wireless routers support WPA/2 and WEP authentication and encryption to ensure data confidentiality on the wireless link.
- Access points implement MAC addresses filtering.
- Wireless network protocol filtering.
- Shield SSID broadcast information.
- Rational allocation of IP addresses.

WEP was the sole security mechanism in the first version of the 802.11 protocol introduced in 1999. WEP supported two methods of authentication from an authorisation standpoint: the open system method and the shared key method. In the first method, the client does not have to provide any credentials for connecting to the AP. The authentication was completed after the exchange of only two messages. Frequently, in such scenarios, the network is protected through means of white-listing specific MAC addresses. WEP also supported a traffic encryption mechanism based on the RC4 algorithm for confidentiality, while the CRC-32 mechanism was employed for message integrity. Confidentiality in WEP relies on a static key, also known as root key. WEP supported two different key sizes, and, as a result, two versions exist, namely WEP-40 and WEP-104. This technology was the first security technology for WiFi communication and is not considered secure anymore.

WPA is the next security technology used for WiFi 802.11x communication security. The scope was to evolve and eliminate previous security problems. The figurative of WPA is the provision of stronger encryption mechanisms, such as the incorporation of the Temporal Key Integrity Protocol (TKIP) and the use of AES encryption. WPA uses central authentication servers (e.g., RADIUS) for user authentication, key management and access control. WPA-PSK is a simplified version of WPA, based on the use of a preshared secret or passphrase among the users.

The WPA2 security technology (IEEE 802.11i) consists of a modification to the original IEEE 802.11 standard aiming to make an even more secure technology for the WiFi protocol. In WPA2, a key hierarchy is used where a single key is placed at the highest level and all subsequent keys are generated from this key. There are two types of keys, depending on the selected authentication method. If the authentication is based on a Pre-Shared Key (PSK), the PSK is the used by the users. In this family of frameworks, there is also the 802.11w. The Robust Security Network Information Elements (RSN IE) field is extended by the two bits Association Query (SA Query) mechanism.

##### ZigBee

In the literature, various security threats against ZigBee have been identified, along with a variety of security controls to mitigate them. Furthermore, the IEEE 802.15.4 standard (used by Zigbee) utilises higher layers to impose MAC layer security. Implemented security mechanisms use AES for symmetric key cryptography and define several security modes (AES-CTR, AES-CBC-MAC, AES-CCM) [181]. There is also the network and application keys. For example, The network key is always transmitted encrypted over-the-air when using the High Security level [147].

There is a feature called wake-on-radio, which is not available from all chip vectors but can prevent an attacker from guessing the activity period of the network. Another useful and practical measure is to use the Non-Volatile-Memory (NVM) of a node to store the nonce states. That could help recover nodes in case of power failure.

For the forwarding attack, Khanji et al. [182] proposed a sequential freshness counter of frames (input or output frame). This counter can reset each time a new frame is sent/received. For the prevention of the non-repudiation security issue, the cluster key could be used. This key is globally common among all nodes in the whole network and, except from non-repudiation, it can provide simplicity in the key management process, improve energy consumption in devices and, of course, refine the security indeed [183].

##### WIA-PA

This protocol provides a secure access authentication mechanism where MAC layer security is based on the IEEE STD 802.15.4-2006. Min et al. [184] propose and implement a security mechanism for WIA-PA and its protocol stack. This architecture utilises a security manager that configures the security strategies. In addition, it uses a security management agent, which is located in the gateway and it provides boundary protection for the field devices. Finally, a device security management module in used in the application sublayer. Their security protocol stack includes the data link layer frame security, the application layer security and the security data flow procedure. The main scope of this implementation is the key management and the data integrity. Wang et al. [125] propose measures for specific known threats.

- For the selective forwarding attack, the WIA-PA network manager is responsible for observing the network device status reports.
- For the Interference attack, WIA-PA uses adaptive frequency hopping to effectively suppress sudden interference and eliminate the frequency selective fading. Some hopping mechanisms for the mitigation of the interference are the Adaptive frequency switch (AFS), Adaptive Frequency Hopping (AFH) and Timeslot hopping (TH).
- For the jamming attack WIA-PA uses AFH, but still there are node energy issues to be solved.
- For the tampering, WIA-PA application layer and the Data link sublayer use message integrity (MIC) to achieve data integrity.

##### ISA 100.11a

ISA 100.11a applies security protection via message authentication and payload encryption for both message categories (single hop, end-to-end messages). The Transport Layer (TL) header controls the end-to-end messages and the single-hop protection takes place on the Doubly Linked List (DLL). The encryption algorithm that is used is the AES-128. In addition, ISA 100.11a defines a set of security keys that are used to ensure secure communication. Symmetric cryptography relies on both communication end points using the same key when communicating securely [185]. Zhang et al. [155] present a security protocol stack to achieve confidentiality integrity and replay protection. Data Link Layer (DLL) frame security, Transport Layer transmission security and security data flow mechanisms are presented in detail.

##### 6LowPAN

6LowPAN security can be implemented at the perception, network and application layer. It uses the IEEE 802.15.4 secondary security level for the protection of the medical data that are transmitted hop-by-hop through the nodes. Additionally, there are a lot of strong mechanisms in the application layer that achieve end-to-end security between the communication of the end devices. Therefore, providing security using cryptographic techniques is required to provide anonymity, confidentiality and integrity to IoT communication devices. In addition, a lightweight version of IPSec can be used as an extension to offer confidentiality and integrity in the IPv4 and IPv6 packets that are transferred between two peers [186].

Benslimane et al. [187] categorise the 6LowPAN security measures in two taxonomies. The first one concerns the communication outside the 6LowPAN network. Inside IoMT systems, especially in case of remote monitoring and treatment, a node may be connected with an external end-device. Thus, the goal is to establish a secure channel for data transmission. Encryption technique like DTLS, host identification technology like HIP and the Internet Key Exchange (IKE) technology are used to compound a secure transportation channel. The second one concerns the “protocols inside communication”. An Intrusion Detection System (IDS) is a tool or mechanism that enables the detection of abnormal activities carried out by an intruder. An IDS can analyse the activity in the network or in the system itself and prevent the infection of the system.

##### LoRa

LoRaWAN uses the AES cryptographic algorithm combined with Cipher-based Message Authentication Code (CMAC) and CTR (Counter Mode Encryption for integrity protection) for encryption. Each LoRaWAN device is personalised with a unique 128 bit AES key (called AppKey) and a globally unique identifier based on EUI-64.

LoRa secures its application payload with end-to-end encryption between the application server and the end-device. Other proposed secure solutions in this layer include HTTPS and VPNs for secure transportation in this layer. For mutual authentication, LoRa uses a “known by the network” key (AppKey). It uses this key to compute an AES-CMAC. We have two keys derived. There is one key to provide integrity protection and encryption of the LoRaWAN MAC commands and application payload and another key for end-to-end encryption of application payload. LoRaWAN traffic is, therefore, protected by using the two session keys. Each payload is encrypted by AES-CTR and carries a frame counter (to avoid packet replay), and a Message Integrity Code (MIC) computed with AES-CMAC (to avoid packet tampering) [129].

#### 4.3.3. Application Layer Mitigations

##### HL7

Data protection is the main target from the security scope because HL7 transmits data that have a high impact. To protect the entire network, instead of just a single application, many institutions are using SSL VPNs and similar solutions. Such solutions allow them to create a secure connection and protect their data from public connections. Deidentification/anonymization helps to protect patient data. There exist various approaches to deidentification, such as deleting directly identifying data, replacing identifying data with artificial identifiers or pseudonyms and suppressing or generalising quasi-identifiers. To conclude, there is a list of measures to protect medical data: (a) the patient does not use personal data such special IDs for medical purpose; (b) encrypt the whole information not selectively; (c) assign access rights for all users; (d) strong password policy, especially for users with admin rights like doctors; and (e) avoid excessive protection and complicated third-party solutions that slow down the network.

##### HTTP

To achieve the desired security level, HTTP is encapsulated over SSL/TLS, to form the secure (HTTPS) version of HTTP communications. The server authentication is usually certificate-based, while for client authentication form-based authentication over TLS is widely used. However, SSL/TLS also supports mutual certificate based authentication for both the client and the server.

##### COAP

A strong authentication technique should be employed for data theft and DoS attacks mitigation. An intrusion system can help the detection of any suspicious activity in the system [83]. DTLS could also be a solution for data security [132].

##### MQTT

For ensuring security, MQTT brokers may require username/password authentication, which is handled by TLS/SSL (Secure Sockets Layer). Note that TLS/SSL is the same security protocol that ensures privacy for HTTP transactions all over the Internet [82].

In Table 5, we summarise the security features, vulnerabilities, attacks and existing security controls for IoMT specific communication protocols.

## 5. Secure IoMT Communications: Threat Landscape and Protocol Comparison

After having analysed the security characteristics of the IoMT specific communication protocols, we review real world medical specific attack scenarios that involve the exploitation of their communication layer. Then, we will provide a comparison of the existing IoMT communication protocols based on realistic medical device use cases.

### 5.1. IoMT Current Threat Landscape

We present some real case attack scenarios based on the device categorisation provided earlier (in Section 3.2). Every malicious action tries to penetrate into a medical device through the communication layer protocols to steal medical information, destroy devices or make healthcare devices useless.

- Attacks on wearable devices: Wearable devices usually collaborate with various devices such as mobile phones through a communication protocol (for example BLE) or with an aggregator (e.g., to collect medical data from various sensors). In this scenario, a smart watch works as a pulse oximeter connected directly with a mobile phone using BLE. The attacker could be in range to pair forcibly with the wearable device. When the user’s mobile phone collects data, the collected data may be accessed by the attacker’s smartphone. This particular attack exploits the vulnerability that the wearable device and smartphone do not authenticate each other at every connection instance. Consequently, the wearable device does not differentiate the real user’s smartphone from the attacker’s. In addition, as we previously presented (see Section 4.3.1), securely updating the authentication credentials, pairing only with authenticated devices and avoiding auto pairing [188,189], are very important measures for avoiding such threats.
- Attacks on infusion pumps: The infusion pump belongs to the treatment devices. It is operated by the patient either remotely or with physical access, thus a doctor or a nurse can handle the device in both ways. Below we present some real case scenarios of proven vulnerable infusion pumps that are used in healthcare areas and a threat that can easily happen. In 2015, the US Federal Drugs Administration (FDA) ceased the use of a specific infusion pump due to a safety breach. Security researchers (Billy Rios in 2014 and Jeremy Richards in 2015) discovered several vulnerabilities for a specific model of Hospira infusion pumps. These vulnerabilities allowed a hacker to tap into the pumps and change the original amount of medication set to dispense. FDA recommended that all hospitals in California and across the country should stop using the vulnerable medical devices [190,191,192]. As infusion pumps can also be handled with physical access, human mistake and physical tampering are also applicable threats. Strong identification and authentication are required to repulse the attacker from accessing the device. In Section 4.3, an RFID identification system and authorisation mechanisms are discussed.
- Attacks on Surgical Robotics: Realistic attack scenarios involve direct attacks against connected surgical robots, or indirect attacks against ambient devices, such as gyroscope sensors, that may affect a surgical operation. The nature of the surgery makes micrometre accuracy highly important. A direct attack to the surgical robot or an indirect attack against the sensors is possible. Perception layer attacks are the main threat for gyroscope sensors. An attacker can make replay attacks, by producing signals to confuse the original gyroscope signals. This attack can cause problems to the mapping of the human body. It may change the coordinates or produce error signals [193]. An attack like this requires close proximity of the attacker with the sensor. So the doctor has to operate from outside the surgery room and the room has to have a lack of monitoring and identification systems. In the case of a direct attack against the surgery robot, the following attacks are possible:
  - *Modification of robot’s intent*. During the transportation of the packets, the attacker may modify them. This action may cause minor malfunctions in the device like unusual robot movements or delays.
  - *Manipulation of the robot’s intent.* The intruder in this case cannot handle the medical device but may affect the feedback of the device like the images and coordinates.
  - *Hijacking.* The intruder takes over the surgical robot.
  In the above scenarios the attackers may act in several ways. First, as network eavesdroppers who collect information. Then as active attackers who also inject small packets. In addition, attackers may act as network mediators (MITM attack) when the in-hospital device stops to communicate directly with the IoMT network. The Address Resolution Protocol (ARP) poisoning attack could be a method to alleviate direct attacks against surgical robotics [194].
- Attacks on monitoring devices: In this case the malicious actor may try to hack a camera or to affect an alarm system. Of course, if someone affects the alarm system of a treatment device and the alarm is switched off for some minutes (even and for seconds), a patient might receive a medicine overdose, resulting in severe consequences. Example of attack patterns involve DoS attacks and SQL injections. Securing from such attacks may require the implementation of advanced Intrusion Detection Systems (IDS) and strong authentication systems [195]. The protocols involved here are mainly from the application layer (COAP, MQTT). The need of early detection is considered highly important [196].

### 5.2. Network Protocol Comparison in IoMT Use Cases

Depending on the type of medical service, the underlying infrastructure and technologies used, the security needs and cost factors in each case, some protocols are considered a safer choice than others. Moreover, the range and the data rate are important characteristics for an IoT network into a healthcare area. An IoMT can be implemented in a small medical centre or in a big hospital. In addition, in some cases, like a live monitoring procedure in a robotic surgery room, we need high data rate. Into an e-prescription case, the low data rate protocols can be useful too. For a detailed comparison of the transmission range and the data rate of typical IoT protocols, we refer to [197].

#### 5.2.1. Short-Range Low-Power Protocols

Relevant research [198] suggests that ZigBee is easier that 6LowPAN when it comes to configuring and planning device energy consumption. On the other hand, 6LoWPAN networks seem to have reduced delays and packet loss rates than ZigBee networks, which makes them better candidates for high availability scenarios involving medical services. ZigBee is also better in device hoping and energy consumption than other alternative protocols like Z-Wave.

Z-Wave has longer optimal range than ZigBee and 6LowPAN due to its sub-1GHz band, but is not an open standard; it supports smaller mesh networks and requires somewhat more power than ZigBee. This communication band also allows Z-Wave to have less interference, since WiFi, BLE and ZigBee utilise the 2.4 GHz band which is more prone to interference. The downside may also be considered its lower data transmission rates.

Comparing 6LowPAN with BLE implementations on medical environments, relevant research [199] suggests that 6LoWPAN is more efficient when using IP-based applications, although there exist connectivity issues when obstacles are in place. Researchers state that 6LoWPAN often lost connectivity if obstacles like clothing were positioned between the transmitter and the receiver.

Considering network connectivity, devices in 6LoWPAN communicate directly with each other, whereas LoRaWAN uses gateways and routers to communicate data to each other. ZigBee on the other hand supports the mesh network topology.

#### 5.2.2. Long-Range Low-Power Protocols

LoRaWAN focuses on wider areas and is made for device communication that can reach up to 10km away using subgigahertz (GHz) radio frequencies.

LTE-M networks can too provide long range (even worldwide) connectivity and have embedded security mechanisms that can support most applications. In addition, the telecommunications infrastructure is robust enough to support most applications. Still, LTE networks have higher power consumption and require specific hardware which are often prohibiting factor for IoMT networks; especially when it comes to low-power monitoring devices are used.

### 5.3. Comparing IoMT Protocols in Medical Use Cases

#### 5.3.1. Use-Case 1: Remote Monitoring and Portable Medical Devices (Ambient, Wearables, Ingestibles, Implantables)

Monitoring devices continuously collect vital signs (such as heart rate and temperature) to allow for the real-time estimation of the patient status. Remote monitoring devices in the IoMT utilise specific sampling rates for continuous monitoring and data are usually securely transferred from on-body or in-body sensors to a data collection unit for processing and storage. Thus, there is a constant exchange of data between on/in-body sensors and remote off-body systems. Similarly, ambient devices such as patient alarm sensors and applications can be used in closed-loop management solutions able to serve multiple solutions to patients, such as in cases of automatic decision making in heart failure and coronary hear disease situations.

Researchers in [200] propose the use of interoperable protocols such as HL7, BLE over ZigBee Health for implementing end-to-end remote monitoring platforms in IoMT and relevant medical devices. However, such implementations require further configuration for explicit acknowledgement requests that are not defined in the IEEE 11073 standard. Still, their preliminary clinical trial results show that ZigBee Health has some advantages over other wireless technologies [200].

Out of all possible remote monitoring devices, ingestibles and implantables require the lowest power consumption. For close device communication, UWB has shown great promise UWB’s power consumption is significantly lower than ZigBee’s and seems to be robust against interference [201]. Still, if IoMT implementations require long distance monitoring, then probably solutions should focus on other technologies such as LTE-M or GSM for robust, long range monitoring.

If implementation instances do not require long range monitoring or IP based solutions, ZigBee Health along with HL7 specifically formatted for medical data currently seems a viable option. Still, if IP applications are required to support software application interconnectivity, implementations of 6LowPAN may be more appropriate to unify technologies and networks including WiFi (802.11). 6LowPAN manages to keep IP connectivity and uses while simplifying its current underlying security and routing mechanisms that were too demanding for small embedded devices [202]. Still, this is the case for short range, in-hospital networks. Mobile solutions from the industry currently focus on using BLE with a smart mobile device that then connects over cellular/GSM to the cloud [203].

#### 5.3.2. Use-Case 2: Drug Delivery Devices—Infusion Pumps

Modern drug delivery devices (most commonly, infusion pumps but also inhalers and injectors) utilise a broad range of connectivity [203]. The most prominent wireless standards used for close range connections involve BLE and ZigBee. These two became the first standards to also be adopted by the Continua Health Alliance consortium for connected medical solutions. Infusion pumps, being in-hospital devices, usually have a constant power supply from a stationary socket. Most certified devices have a battery life of approximately 2 h to cope with power outages. Most pumps connect over wired/USB Ethernet and/or WiFi modules [203]. Thus, typical IEEE 802.11.x and IEEE 802.15.x security aspects apply.

As far as wired connections go, many infusion pumps implement custom protocols over IP or directly use TCP/IP connections over IPv4 to transfer data for minimal packet loss and high availability. Still, being able to transfer and offering multiple closed-loop services, such connections should opt to minimise power consumption while, at the same time, follow standard security measures for Ethernet and IEEE 802.11 devices.

Standardisation bodies mostly refer to WLAN and WiFi technologies for wireless communication of medical pumps and propose security controls commonly used with such technologies [204]. This includes strong authentication, SSL pinning, Multi-Factor Authentication (MFA) and AES encryption. Remote access should only be allowed through a VPN client at the network layer. Wireless connections should include WPA2 with Extensible Authentication Protocol (EAP)-TLS for protecting data exchange. Another important note here is that infusion pumps should not include hardcoded encryption or authentication keys in their code: a weakness often overlooked by vendors.

#### 5.3.3. Use-Case 3: Large Institutional Medical Devices

Large, stationary institutional medical devices such as Surgical Robotics, MRIs, digital X-Ray and other medical imaging or treatment machines prioritise data integrity and availability over flexibility and low power consumption. Such devices often use application layer protocols such as HL7 over wired Ethernet connections or wireless WiFi [203,205]. As such, underlying network protocols focus on constant availability and data integrity, rather than low power consumption and connection flexibility. Common IoMT protocols like ZigBee, 6LowPAN and BLE are not used due to their low bandwidth capabilities (imaging often results in high-resolution, big data files). Instead, typical implementations use at least 1Gb Ethernet over MAC layer and IEEE 1588 solutions, PC/SMBus and/or PCIe connection slots and USB [206], although some solutions exist that utilise ZigBee alongside Ethernet for wireless communications [203]. Similarly to drug delivery devices, such installations should focus on a constant high power supply through generators and typical IT security controls for protecting the network and application layers.

## 6. Challenges and Open Issues

Even though IoT devices and networks increase their security level as their underlying technologies come of age, still numerous security issues exist when it comes to protecting patient health data. Medical services have increased risks for the population. This, coupled with the lack of a uniform standardisation scheme and the diversity of technologies used in such networks makes protecting medical services an even more daunting task. Future efforts should address this technical dissonance and examine the common characteristics used in medical services to develop an overall scheme for implementing IoMT services. This will require a good understanding of medical devices and services so as to ensure that their elevated requirements are met in all cases.

### 6.1. Organisational Challenges

International bodies and relevant state actors should work towards the mapping of modern medical services onto current technological trends and propose unified approaches in tackling the security of IoMT networks. This will require accuse coordination with the industry in order to catalog the security needs together with device restrictions, since the medical devices are already subject to multiple safety and development standards. The potential future standardisation of IoMT networks must take into consideration all the current safety requirements and standardisation processes in place for medical devices. As such, IoMT network protocols will have to be designed with specific restrictions in mind to embrace the current legal and operational frameworks.

### 6.2. Data Privacy and Technical Issues

Each IoMT protocol has its own strengths and weaknesses. Still, due to the elevated risk of medical services, some issues will require more attention than others.

1. Key management remains an open issue for medical networks, especially when it comes to wearable devices or ad-hoc connections. Current solutions may partially mitigate most issues but, still, there exists no unified approach able to tackle security concerns both for Wireless Sensor Network (WSN) and ad-hoc networks to the extent required by medical services.
2. The technical heterogeneity of IoMT networks paves the way for numerous attacks. Currently, there exists no unified standardisation process or protocol able to mitigate the security issues introduced by such heterogeneous technologies. This is true for all the IoT networks, not only those implemented for the medical sector [207]. Even worse, medical networks have even more stringent rules and require stronger security than common IoT networks.
3. Out of all the documented attacks and exploits, DoS remains the most frequent type of attack applicable in IoT networks. Since medical devices and services rely heavily on their availability, such attacks can be considered extremely dangerous for patients. Unavailability of medical devices (either through lack of connectivity or lack of power) can lead to elevated risks or even patient death, if certain circumstances are met.
4. The existing protocols mostly inherit the pros and cons of current WSN standards like IEEE 802.15.x. Currently it is difficult to balance secure authentication mechanisms with power consumption. This is extremely important for the medical sector, since computation and communication overhead in the IoMT can result in power depletion; a state that can be proven fatal when it comes to medical services. Trust management and node management are also important when it comes to protecting the confidentiality of medical data. Numerous attacks on IoMT either use a man-in-the-middle node or exploit the trust relations to leak sensitive data.
5. Data confidentiality is of utmost importance when it comes to protecting medical services. As such, the use of a horizontal rule when it comes to encrypting data in the IoMT is mandatory. This is something that should also be addressed at a legal level, where possible extensions or an explanatory circular on the General Data Protection Regulation (GDPR) can specifically be used to address encryption schemes for modern medical networks.

## 7. Conclusions

Due to their inherent complexity and diversity, IoMT networks cannot adopt a single network protocol for all possible implementations. Different medical environments have different needs in processing, communication and device connectivity. For example, wearable devices on ad-hoc networks need increased node privacy measures while maintaining flexibility and low power consumption. On the other hand, network protocols in large infrastructures such as hospitals require increased overall security, common data patterns while being more lenient on power consumption and flexible connectivity.

The rapid adoption of IoT in the medical sector suggests that the integration of WSN and sensor networks in medical services will continue to increase both in complexity and size. This article reviews recent research on IoT protocols and presents their capabilities along with potential security issues when these protocols are implemented on medical IoT networks. First, we introduced a classification of communication protocols per medical device types, as utilised in the health care sector. Then, we systematically catalogued and analysed their security challenges and proposed solutions applicable to medical environments. Contrary to previous IoT surveys, this article focuses on the medical sector and the prevalence of IoT devices in medical clinics, hospitals and custom networks populated by wearable devices that monitor and regulate patient information. We believe that this paper will be a useful point of reference for future researchers interested in analysing network protocol security for medical devices. 

## Figures and Tables

**Table 1:** Key words used during the search phase.

Scientific Literature	Grey Literature
**Keywords for the first stream**: TITLE (IoMT OR “Internet of Medical Things” OR “medical” OR “healthcare” OR implantable OR implanted AND protocol OR “communication protocol”) **Keywords for the second stream**: TITLE (IoT OR “Internet of Things” AND security OR vulnerability OR threat OR attack OR control)	Medical, healthcare, communication protocols

**Table 2:** A summary of Perception and Network layer IoMT Communication Protocols.

	Layer	Relevant Literature	Frequency	Data Rate	Range	Power Cons/Ion	Definition
**IoT Protocols**							
IrDA(Infrared)	Perception-Network	[12,15,26,62]	850–900 nm	14.4 Kbps	1 m	na	Protocol
RFID	Perception	[12,15,45,52,63,64,65]	13.56 MHz	106–424 Kbps	20 cm	Very Low	Technology
NFC	Perception-Network	[7,15,36,66,67]	13.56 MHz	106–424 Kbps	20 cm	Very Low	Technology
Bluetooth/BLE	Perception-Network	[7,12,13,15,37,68,69,70]	2.4 GHz	1, 2, 3 Mbps	80–90 m	Low	Technology
ZWave	Perception-Network	[18,71]	sub GHz	40 kbps	30 m	low	Protocol
UWB	Perception	[7,12,15,37]	3.1–10.6 GHz	53–480 Mbps	10 m	Very Low	Technology
WiFi	Network	[7,37,47,63,72]	2.4 GHz, 5 GHz	0.1–54 Mbps	80 m	Medium	Protocol-Standard (ISO)
ZigBee	Perception-Network	[7,13,36,37,47,64,69,70,73]	2.4 GHz	250 kbps	100 m	Low	Protocol
WIA-PA	Network	[52]	na	na	na	na	Standard
ISA100.11a	Network	[52]	na	na	na	na	Technology
6LoWPAN	Network	[7,13,36,37,45,47,52,63]	2.4 GHz	na	na	na	Technology-Set of standards
LoRa WAN	Network	[7,60,61]	sub GHz	50 kbps	3–4 km	Low	Protocol

**Table 3:** Internet of Medical Things (IoMT) application protocols.

	Layer	Relevant Literature	Standards	Encoding Format	Architecture	Header/Message
**IoT Protocols**						
COAP	App	[36,47,70,86,87,88]	IETF, Eclipse foundations	Binary	Client-Server, Broker	4 Byte/Small
MQTT	App	[47,65,68,85,86,89]	OASIS, Eclipse foundations	Binary	Client-Broker	2 Byte/Small
HTTP	App	[7,47,70,73,85,89]	IETF, W3C	Text	Client-Server	Undefined, Large
HL7	App	[87]	ANSI/ISO/HITSP/CDA/EHR/FHIR/CCOW	UTF-8/16/32, ISO 8859-1	Client-Server	402/1039 bytes

**Table 4:** A categorisation of the most common IoMT Devices.

Physiologic Monitoring	Medical Treatment	In-Hospital Connected	Ambient	Additional Devices
Motion sensors	Infusion pumps	MRI	Identification devices	Coordinators
Blood glucose device	ICPs	X-rays	Gyroscope sensors	Network devices
Blood pressure device	Cardiac rhythmic management	Surgical Robotics	Motion sensors	End user devices
Temperature sensor	Smart medical capsules	Prosthetic using Surgical Robotics	Vibration sensors	Databases
Pulse oximetry			Monitoring devices	Servers
Pacemakers			Alarm devices	
EEG sensors			Implantable device Charger	
ECG sensors				
Respiratory rate sensors				
Muscle activity sensors				
Implantable devices				
Pill-line sensors				
Aggregators				

**Table 5:** IoMT protocols security features, vulnerabilities, attacks and possible controls.

Protocol	Security Features	Vulnerabilities	Attacks	Controls	
IrDA(Infrared)	No embedded security controls.	Detect reflected infrared-light and filtering out the surrounding ambient noise.	Eavesdrop attack.	Physical security controls.	
RFID	Embedded data are unprotected and read only.	Active (continuously transmitting) and passive (electromagnetic field) RFID systems suffer from weaknesses.	Side channel attack.	Authentication-hash based protocols, encryption functions.	
NFC	SSE, SCH, 3 modes of operation: Read/Write, Peer-to-Peer and Card Emulation Mode.	Data exchange in close proximity, PICC emulations in protocol challenge-response requests.	Near proximity, MITM, DoS, Modification attacks.	Architecture and the distance limitations, secure channel with a standard key agreement protocol.	
Bluetooth/BLE	Secure simple pairing (SSP), Connectivity issues over obstacles	Encryption of the payload and not of all the entire packet, matching the connection’s frequency hops and then capturing data in that frequency range, address verification, PINs.	Sniffinig, DoS, MITM, Brute-Force, device duplication attacks.	AES-CCM, 4-byte MIC module, AES-128	
ZWave	AES encryption with three shared keys.	Does not enforce a standard key exchange protocol, Z-Wave devices implicitly trust the source and destination fields of794the MPDU frame.a malicious node can assigned by the controller.	Key Reset, impersonation, node spoofing, BlackHole attacks.	AES-128 with three shared keys.	
UWB	LRP/HRP secure ranging schemes, size of the UWB symbol.	Long symbols length, wrong access control configuration or power failure.	ED/LC, Same-Nonce attack.	Localization and distancing protocols secure the range between nodes.	
WiFi	WPA2, SSID hiding, MAC filtering and static IP addressing, Connectivity issues over obstacles	Lack of granular device authentication, weakness against denial of service, limited protection of service integrity.	DoS, Replay, Channel collision, Spoofing attacks.	WPA, WPA2 capability, 128-bit WEP authentication.	
ZigBee	128-bit AES with pre-share keys, frame-protection mechanisms, essential key(encryption in network layer), global link key and unique link key(App layer), Connectivity issues over obstacles	Utilizing insecure key transportation for pre-shared keys, ACKs have no integrity checks, insufficient registration of network keys, the lack of verification in PAN IDs.	Installing default link keys or sending security headers in clear text on auxiliary frames, looding that causes DoS, euses of Initiation Vectors which may lead to key compromise, energy-consuming attacks.	AES for symmetric key, AES-CTR, AES-CBC-MAC, AES-CCM, Use the Non Volatile Memory of the node to store the nonce states, Key management algorithm.	
WIA-PA	Join-key shared between device and security manager.	Lack of public key encryption algorithm, no intrusion prevention, no broadcast key, The first request is not encrypted.	Sybil, DoS, wormhole, Jaming , traffic analysis attack.	AFS, AFH, TH, MIC.	
ISA100.11a	Linchpin, AES-128, time limitations	Requires some special conditions to be implemented in a secure path.	Sniffing, Spoofing, Replay attacks and Data falsification.	AES-128 on TL header.	
6LoWPAN	AES cipher suit, ESP, IKEv2, DTLS, Connectivity issues over obstacles	IP network, radio signal of implementations, Unchanged nodes address, fragmentation mechanism.	Use of malicious intermediary network nodes, Signal jamming, traffic analysis, attackers selectively prevent correct packet reassembly.	DTLS, HIP, IKE, cryptographic techniques.	
LoRa WAN	128-bit application session key (AppSKey), AES.	Resetting frame counters without re-keying, caching and replay of ACK packets, transmit falsified gateway beacons to repeatedly wake up sensors, utilize a dictionary of pastmessage.	Replay attacks, recovery of passwords, malicious message modification, battery exhaustion and DoS.	AES-CMAC, AAES-CTR, MIC.	
HL7	No built-in security.	Message sources are often not validated by default, ize of HL7 messages is often not validated.	Spoofing or integrity attacks, Flooding attacks.	SSL, VPN.	
HTTP	Basic-Digest authentication.	Data transfer is not encrypted, Get request.	Evasedropping- theft- breach and manipulation, flooding attacks	SSL/TLS(HTTPS)	
COAP	NoSec- SharedKey -MultiKey- Certificate mode.	Proxies having to decide if DTLS implementation will be multi-cast or uni-cast message.	Parsing, Cache, amplification, spoofing. Cross-protocol attacks.	DTLS, Strong authentication technique.	
MQTT	Four-way handshake mechanism.	No embedded data encryption mechanism, IP broker (sometimes is unsecure).	Traffic analysis, Port Obscurity, Botnet Over MQTT.	SSL/TLS	
